# Genetic diversity of murine norovirus populations less susceptible to chlorine

**DOI:** 10.3389/fmicb.2024.1372641

**Published:** 2024-04-30

**Authors:** Aken Puti Wanguyun, Wakana Oishi, Andri Taruna Rachmadi, Kazuhiko Katayama, Daisuke Sano

**Affiliations:** ^1^Department of Frontier Sciences for Advanced Environment, Graduate School of Environmental Studies, Tohoku University, Sendai, Japan; ^2^Department of Civil and Environmental Engineering, Graduate School of Engineering, Tohoku University, Sendai, Japan; ^3^Environmental and Food Virology Laboratory, Institute of Environmental Science and Research Ltd (ESR), Kenepuru Science Centre, Porirua, New Zealand; ^4^Laboratory of Viral Infection I, Department of Infection Control and Immunology, Ōmura Satoshi Memorial Institute & Graduate School of Infection Control Sciences, Kitasato University, Tokyo, Japan

**Keywords:** chlorine, disinfection, genetic diversity, murine norovirus, less susceptible

## Abstract

High genetic diversity in RNA viruses contributes to their rapid adaptation to environmental stresses, including disinfection. Insufficient disinfection can occur because of the emergence of viruses that are less susceptible to disinfection. However, understanding regarding the mechanisms underlying the alteration of viral susceptibility to disinfectants is limited. Here, we performed an experimental adaptation of murine norovirus (MNV) using chlorine to understand the genetic characteristics of virus populations adapted to chlorine disinfection. Several MNV populations exposed to an initial free chlorine concentration of 50 ppm exhibited reduced susceptibility, particularly after the fifth and tenth passages. A dominant mutation identified using whole-genome sequencing did not explain the reduced susceptibility of the MNV populations to chlorine. Conversely, MNV populations with less susceptibility to chlorine, which appeared under higher chlorine stress, were accompanied by significantly lower synonymous nucleotide diversity (π_S_) in the major capsid protein (VP1). The nonsynonymous nucleotide diversity (π_N_) in VP1 in the less-susceptible populations was higher than that in the susceptible populations, although the difference was not significant. Therefore, the ability of MNV populations to adapt to chlorine was associated with the change in nucleotide diversity in VP1, which may lead to viral aggregate formation and reduction in chlorine exposure. Moreover, the appearance of some nonsynonymous mutations can also contribute to the alteration in chlorine susceptibility by influencing the efficiency of viral replication. This study highlights the importance of understanding the genetic characteristics of virus populations under disinfection, which can contribute to the development of effective disinfection strategies and prevent the development of virus populations less susceptible to disinfectants.

## 1 Introduction

Human noroviruses cause acute gastroenteritis and pose a significant public health risk (Duizer and Koopmans, 2006). Approximately 18% cases of acute gastroenteritis outbreaks in all age group were caused by norovirus infections worldwide (Ahmed et al., 2014; Lopman et al., 2016). Furthermore, >50% foodborne diseases are caused by viruses, resulting in approximately 200 million infections and over 200,000 deaths among children annually in the USA (Debbink et al., 2014). Noroviruses are single-stranded positive-sense RNA viruses of the *Caliciviridae* family, and their approximately 7.5 kilobase-long genome comprise mainly three open reading frames (ORFs); however, the murine norovirus (MNV), which harbors four ORFs, is an exception (Carstens, 2010; McFadden et al., 2011). Noroviruses can be classified into ten genogroups (GI–GX) based on the amino acid sequence diversity of the major capsid protein (Chhabra et al., 2019). GI and GII are the primary pathogenic genogroups in humans, with GII contributing to approximately 90% of the norovirus outbreaks (Weinberg, 2019).

Noroviruses can be transmitted via the fecal–oral route, either via contact with an infected person or via exposure to food, water, or environmental surfaces contaminated by infected vomit (Marks et al., 2000; Lysén et al., 2009; Verhoef et al., 2015). Furthermore, most fecal–oral-transmitted viruses, such as noroviruses, are shed in the fecal matter, are highly stable in aquatic environments, and are often found at high concentrations in wastewater (Hellmér et al., 2014). Viral exposure can occur from the extensive utilization of water bodies for human activities, such as irrigation and leisure activities (Dias et al., 2019). Therefore, appropriate strategies for virus removal from wastewater are crucial for minimizing the viral load in aquatic environments. Effective disinfection is essential during the final phase of wastewater treatment.

Chlorination is the most commonly used method for microbial inactivation owing to its cost-effectiveness (Collivignarelli et al., 2018). Chlorination inactivates viruses by damaging their nucleic acids and proteins, which interfere with viral genome replication (Qiao et al., 2022). The concentration of the disinfectant, exposure time, pH, temperature, and virus type are the essential factors determining the effectiveness of the disinfection (Rachmadi et al., 2018; Lin et al., 2020; Kadoya et al., 2022; Oishi et al., 2022). Numerous studies have highlighted the variability in disinfection sensitivity among different virus types and even within specific serotypes and genotypes. Previous studies have demonstrated varying susceptibilities of MNV to several disinfection methods (Rachmadi et al., 2018; Oishi et al., 2022). Rachmadi et al. revealed that compared to control populations, MNV populations repeatedly exposed to chlorine exhibited reduced sensitivity to the disinfectant owing to a unique amino acid replacement in the minor capsid protein (Rachmadi et al., 2018). Other inactivation studies on several viruses have revealed that the Faulkner strain of coxsackievirus B5 was more resistant to chlorine than adenovirus, enterovirus, or MNV under similar inactivation conditions (Black et al., 2009; Cromeans et al., 2010; Kahler et al., 2010). High genetic diversity because of high mutation rates and error-prone replication mechanisms contributes to the differences in sensitivity among RNA viruses (Sanjuán, 2008). Consequently, RNA viruses can evolve rapidly and adapt to various environmental conditions. Certain genetic variations within the viral genome can cause resistance or reduce the sensitivity to disinfectants (Kadoya et al., 2022; Oishi et al., 2022). However, the genetic characteristics that contribute to alterations in susceptibility to particular disinfectants are not understood.

This study aimed to understand the genetic characteristics of virus populations adapted to chlorine disinfection. We performed an experimental adaptation using MNV as surrogates for human norovirus. Subsequently, we examined the disinfectant susceptibility and genetic characteristics of the whole genome of all MNV populations using next-generation sequencing (NGS). Our observations will contribute to the development of effective disinfection strategies and prevent the development of virus populations less susceptible to disinfectants.

## 2 Materials and methods

### 2.1 MNV samples and cell lines

The MNV S7 propagations were produced in RAW 264.7 murine “macrophage-like” cells (ATCC TIB-71) in T75 flasks (Nunc™ EasYFlask™ Cell Culture Flasks, ThermoFisher Scientific) containing approximately 80% confluent cell monolayer according to standard protocols (Gonzalez-Hernandez et al., 2012). The RAW264.7 cells were cultured in Dulbecco’s modified Eagle’s medium supplemented with 0.1% (w/v) NaHCO_3_, HEPES, 10 mM nonessential amino acids, 100 mg/mL penicillin, 100 U/mL streptomycin, 2 mM L-glutamine, and 10% fetal bovine serum (Gibco). Subsequently, the cells were incubated in a 5% CO_2−_humidified atmosphere at 37°C.

### 2.2 *In vitro* serial passages

**Figure 1:**
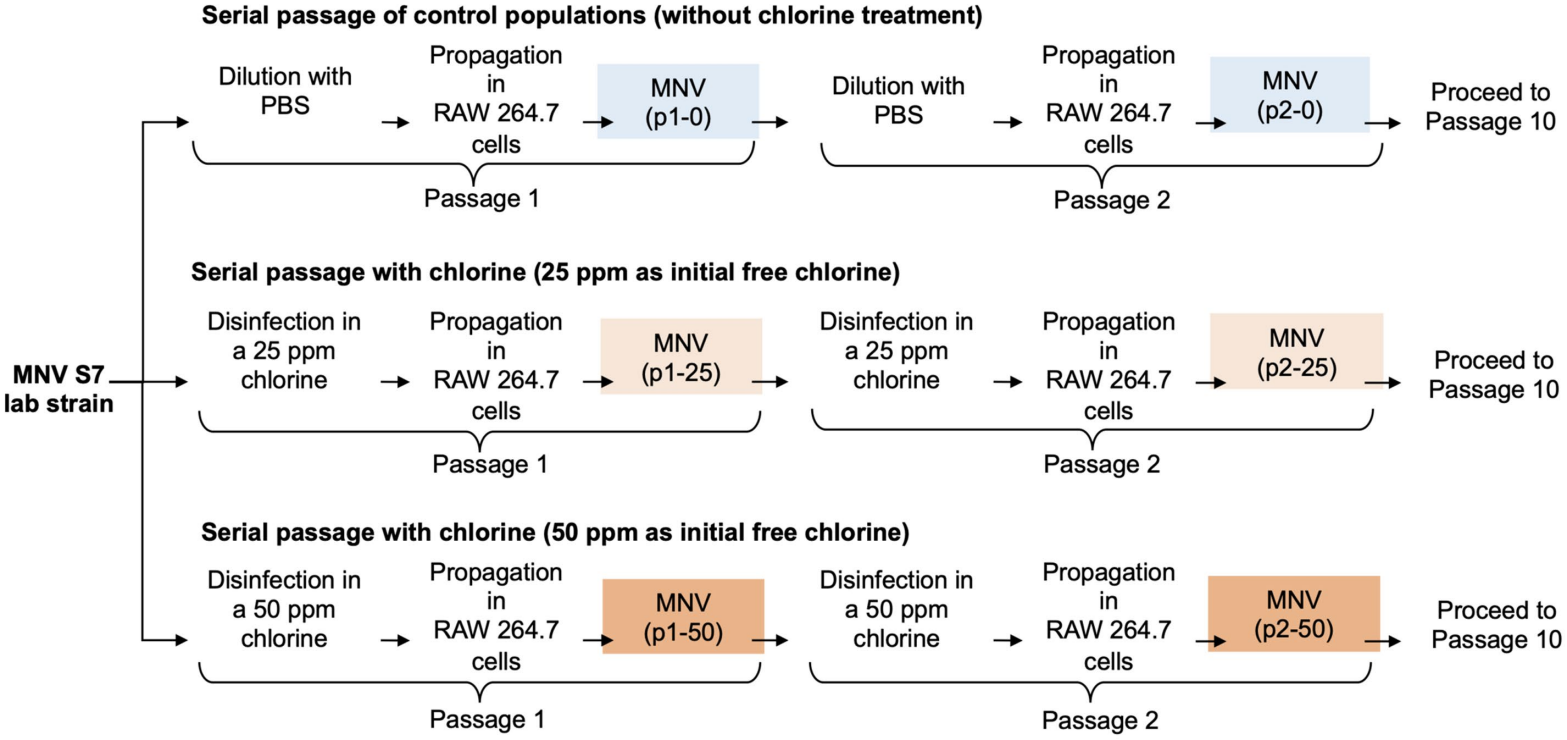
Schematic representation of the three conditions during serial passage experiments. In total, ten passages were performed under each condition with two replications (round 1 and round 2).

Serial passages of MNV were performed as described previously (Rachmadi et al., 2018). Wild type MNV S7 was grown to a titer of 10^7^ TCID_50_/mL in RAW 264.7 cell monolayers to prepare a virus stock. This virus was used for serial passage experiments under several conditions for the control and chlorine-treated populations. MNV populations obtained via dilution from the *x*th passage were treated using chlorine solution with initial free chlorine concentrations of 0, 25, and 50 ppm and were named p*x*-0, p*x*-25, and p*x*-50 ppm virus, respectively (*x* = 1, 2, 3, 4, 5, 6, 7, 8, 9, 10). The entire serial passage experiment of MNV was performed in duplicate (rounds 1 and 2) for each population to assess the reproducibility of the responses to chlorine (Figure 1). Furthermore, as the susceptibility of the RNA virus population to disinfectants may differ, the virus population in each passage was analyzed separately for the first and second rounds (Rachmadi et al., 2018; Kadoya et al., 2022; Oishi et al., 2022).

The chlorine solution was prepared by adding a hypochlorite solution to Dulbecco’s phosphate-buffered saline (PBS) and was analyzed using a DR900 colorimeter with N, N-diethyl-p-phenylenediamine reagent (TOA DKK, Tokyo, Japan). p*x*-25 and p*x*-50 ppm MNV suspensions (10^7^ TCID_50_/mL in 0.5 mL) were exposed to 4.5 mL chlorine solution for 2 min at room temperature (around 22°C) to achieve approximately a 3-log_10_ inactivation, which is the minimum required level of inactivation after disinfection (USEPA, 2020). Fifty microliters of a 1% (v/v) sodium thiosulfate solution was added to neutralize chlorine. Simultaneously, 0.5 mL of p*x*-0 ppm MNV suspension was diluted with PBS to prepare 10^3^ TCID_50_/mL solution, and 50 μL of 1% (v/v) sodium thiosulfate solution was added. MNV was grown until the RAW 264.7 cells were 80% confluent. RAW 264.7 cells with approximately 10^−5^ multiplicity of infection were cultured and incubated in the presence of 5% CO_2_ at 37°C for approximately 72 h. The viral cell suspension was lysed via three freeze–thaw cycles to release the virus particles from the cells. The supernatant was then centrifuged at 10,000 × *g* for 30 min at 4°C and filtered using a 0.45 μm polyvinylidene difluoride membrane filter to remove the cell fractions. The propagated MNV samples were stored at −80°C until subsequent analysis.

### 2.3 Chlorine sensitivity assay

The chlorine sensitivity of the MNV populations was assessed based on the reduction in viral titers following exposure to an initial free chlorine concentration of 50 ppm. One hundred microliters of MNV suspension were mixed with 900 μL chlorine solution with 50 ppm free chlorine and incubated for 2 min. Twenty microliters of a 1% (v/v) sodium thiosulfate solution was used to neutralize the chlorine. The chlorine sensitivity assay was performed in triplicate for each sample.

MNV infectivity was reanalyzed using TCID_50_ (50% tissue culture infectious dose) assays. Three-fold serial dilutions of the virus sample were performed in a 0.5 mL tube containing medium. The diluted viral suspension was transferred to a 96-well plate, and the RAW 264.7 cell suspension was inoculated into each well in 1:1 ratio. Then, the plates were incubated in the presence of 5% CO_2_ at 37°C for 48 h. Next, the cytopathic effects were analyzed using light microscopy. The TCID_50_ values were calculated using the Spearman and Kärber method (Hierholzer and Killington, 1996) and used to calculate the log_10_ reduction value (LRV), which is the ratio of the virus titer after disinfection at time *t* to the initial virus titer before disinfection at time 0 (log_10_ ( Nt/N0 )).

### 2.4 Library preparation and whole-genome sequencing

After serial passaging, the resulting virus populations were characterized based on their complete genome sequence. Viral RNA was extracted from the MNV suspension using the QIAamp viral RNA mini kit (Qiagen, Hilden, Germany). rRNA was depleted using a NEBNext rRNA depletion kit (human/mouse/rat; New England, Ipswich, MA, USA). Subsequently, the NEBNext Ultra II RNA library prep kit for Illumina was used for library preparation according to the manufacturer’s protocol. A barcode sequence was added to each sample using NEBNext multiplex oligos from Illumina. Subsequently, the AMPure XP magnetic beads (Beckman Coulter, Pasadena, CA, USA) were used for library purification. Library concentration and size distribution were analyzed using an Agilent Bioanalyzer, HS DNA assay kit, and a Qubit 2.0 fluorometer (Invitrogen, Carlsbad, CA, USA). The purified library was quantified using the NEBNext Library Quant kit of Illumina. The libraries were sequenced with paired-end 150-bp reads on an Illumina NovaSeq 6,000 (Illumina, San Diego, CA, USA).

### 2.5 Sequence alignment

The CLC Genomics Workbench Software version 10.1.1 (CLC Bio, Aarhus, Denmark) was used to investigate FASTQ-formatted sequence data. The forward and reverse index primers were trimmed using the *Trim Sequences* function. The reference sequence for MNV S7 PP3 was obtained from the NCBI (GenBank accession number: AB435515.1). The *Reference Mapping* function of the CLC Genomics Workbench was used to assemble the trimmed reads into the reference sequence, which was then realigned using the *Local Realignment* function. Next, BAM files were exported from the CLC Genomics Workbench and examined using SAMtools 1.16.1 to determine the sequence coverage of each sample (Danecek et al., 2021). The alignments of all virus sequence data had sufficiently deep coverage; therefore, variant information was provided with high confidence (Supplementary Figure S1). The consensus sequence was extracted from the ancestor MNV populations in the experimental adaptation and was used as a reference sequence for detecting variants using the *Extract Consensus Sequence* function.

### 2.6 Detection of single nucleotide polymorphisms (SNPs)

The frequency of SNPs in the collected samples of the MNV populations was calculated using the *Low Frequency* Var*iant Detection* function. The MNV S7 PP3 genome sequence (GenBank accession number: AB435515.1) was used as the reference sequence, and the error rate threshold value was set at 1%. SNPs with fewer than 10 coverage depths were separated using the CLC Genomics Workbench in the default setting. Whether the SNPs changed the amino acid sequence was also analyzed using the *Amino Acid Changes* function for three ORFs, encompassing ORF1 (nt 6–5,069), encoding a non-structural polyprotein, ORF2 (nt 5,056–6,681), encoding a major capsid protein (VP1), and ORF3 (nt 6,681–7,307), encoding a minor capsid protein (VP2) (Barron et al., 2011). AlphaFold was used to construct the three-dimensional structure of the protein sequences (ORF2 and ORF3) and UCSF ChimeraX version 1.7.1 was used for visualization (Goddard et al., 2018; Jumper et al., 2021; Pettersen et al., 2021; Meng et al., 2023).

### 2.7 Analysis of genetic diversity

The genetic diversity of the control and treated populations was analyzed using different parameters, such as the mean of pairwise nucleotide diversity (π) (Nelson and Hughes, 2015), Shannon entropy (Pan and Deem, 2011), and Tajima’s statistic (*D*) (Tajima, 1989). Site-specific nucleotide diversity was estimated using SNPGenie (Nelson et al., 2015). π is the average number of pairwise differences per site in a population of sequences that was calculated using the following formula:

(1) $$\pi =\overset{p}{\underset{i=1}{\sum }}Ni/L$$

(2) $$Ni=\frac{AiCi+AiGi+AiTi+CiGi+CiTi+GiTi}{\left(k_{i}^{2}-ki\right)/2}$$

where *p* is the site of polymorphism, *Ni* is the nucleotide difference proportion at the *i*th site, *L* is the sequence length, *ki* is the coverage of the sequence at the *i*th site, and *Ai*, *Ci*, *Gi*, and *Ti* are the sums of the four bases at the *i*th site (Nei and Gojobori, 1986; Nelson et al., 2015; Kadoya et al., 2022). The nucleotide diversity in nonsynonymous and synonymous coding sites (π_N_ and π_S,_ respectively) was determined separately using the same formula as the nucleotide diversity. Only *Ni* was adjusted based on the pairs of synonymous or nonsynonymous substitutions (Nelson et al., 2015). Additionally, we measured the ratio of the rates of nonsynonymous and synonymous substitutions (π_N_/π_S_) to evaluate the action of natural selection. π_N_/π_S_ values <1, 1, and > 1 indicate the purifying, neutral, and positive selections, respectively (Nelson et al., 2015). This approach assumes that synonymous substitutions are selectively neutral and provides a baseline for fixation (Raghwani et al., 2016).

### 2.8 Nucleotide substitution rate

The sequences were aligned using MUSCLE to estimate the nucleotide substitution rate. The BEAST2 software, based on the Bayesian Markov chain Monte Carlo method, was used to calculate the nucleotide substitution rate in each genome segment (Bouckaert et al., 2019). Nucleotide substitution rates were expressed as substitutions/site/passage in three different MNV genomic regions in the control and chlorine-treated populations (25 and 50 ppm of initial free chlorine exposure). The BEAST files were run according to a log-normal relaxed clock under coalescent models with the general time-reversible model as a substitution model. The output files were visualized and examined using the TRACER program (Rambaut et al., 2018).

### 2.9 Statistical analysis

Statistical analysis was performed using the Graph Pad Prism software (Version 9.5.0 (525), 2022). The Mann–Whitney *U* test was used to confirm the statistical significance of the differences between the two groups. Additionally, the Wilcoxon signed-rank test was utilized to assess whether there was a significant difference between paired observations based on the median difference. All statistical tests were two-tailed, with *p* < 0.05 considered to be statistically significant. The results were visualized using RStudio (Version 2022.02.3) and Python 3.11.

## 3 Results

### 3.1 Emergence of less-susceptible MNV populations at higher chlorine concentrations

**Figure 2:**
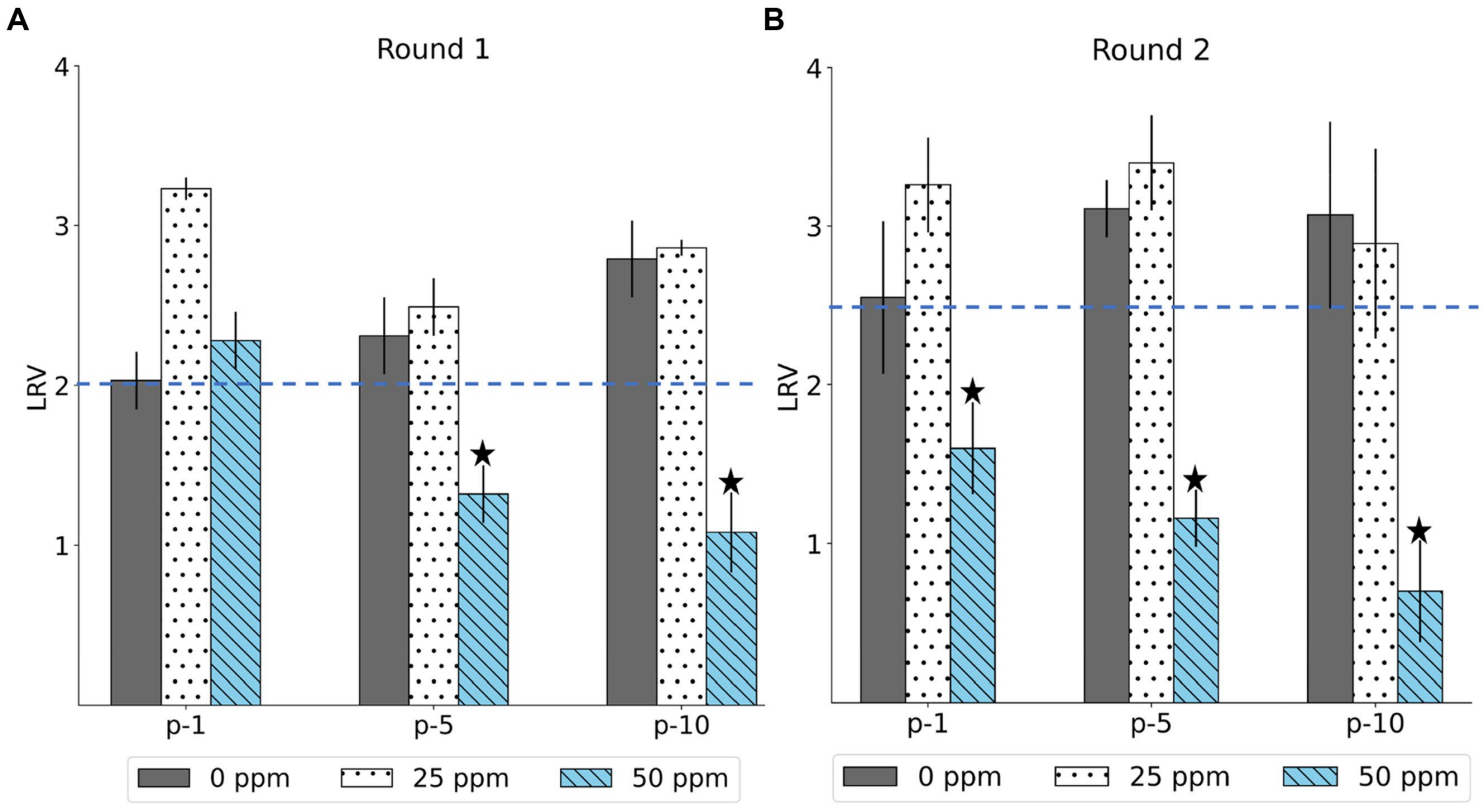
The log reduction values (LRV) of MNV populations were derived from the first, fifth, and tenth passages (p-1, p-5, p-10) in the first **(A)** and second **(B)** rounds. The blue dashed line represents the median LRV of control population (0 ppm) in the first passage of each round. The less-susceptible populations are indicated by star symbols above the bars. Each bar represents the mean LRV from three replicates with standard deviation.

The LRV of the MNV populations derived from the first, fifth, and tenth passages in both the control and treated virus populations is shown in Figure 2. The chlorine susceptibility of MNV populations adapted to an initial free chlorine concentration of 50 ppm was lower than other MNV populations. The Wilcoxon signed-rank test was utilized to identify the MNV population with lower susceptibility to chlorine exposure. We considered the following null hypothesis: the LRV of the treated MNV populations was equivalent to the median LRV of the control populations in each round. As results, five treated MNV populations had lower median values of LRV than the control populations. However, the difference was not statistically significant (*p* > 0.05). These five MNV populations were exposed to an initial free chlorine concentration of 50 ppm obtained in the fifth and tenth passages of the first round and the first, fifth, and tenth passages of the second round. These populations were considered less susceptible. The remaining virus populations were defined as susceptible. The treated MNV populations exposed to 50 ppm of initial free chlorine in round 2 showed reduced susceptibility to chlorine earlier than the treated MNV populations in round 1. One possibility is the existence of subpopulations within the initial viral populations of round 2 that inherently exhibited low susceptibility to chlorine. Upon exposure to 50 ppm of initial free chlorine, these subpopulations might be rapidly enriched, potentially leading to an earlier reduction in susceptibility as the less susceptible subpopulations became dominant.

### 3.2 Nucleotide diversity

**Figure 3:**
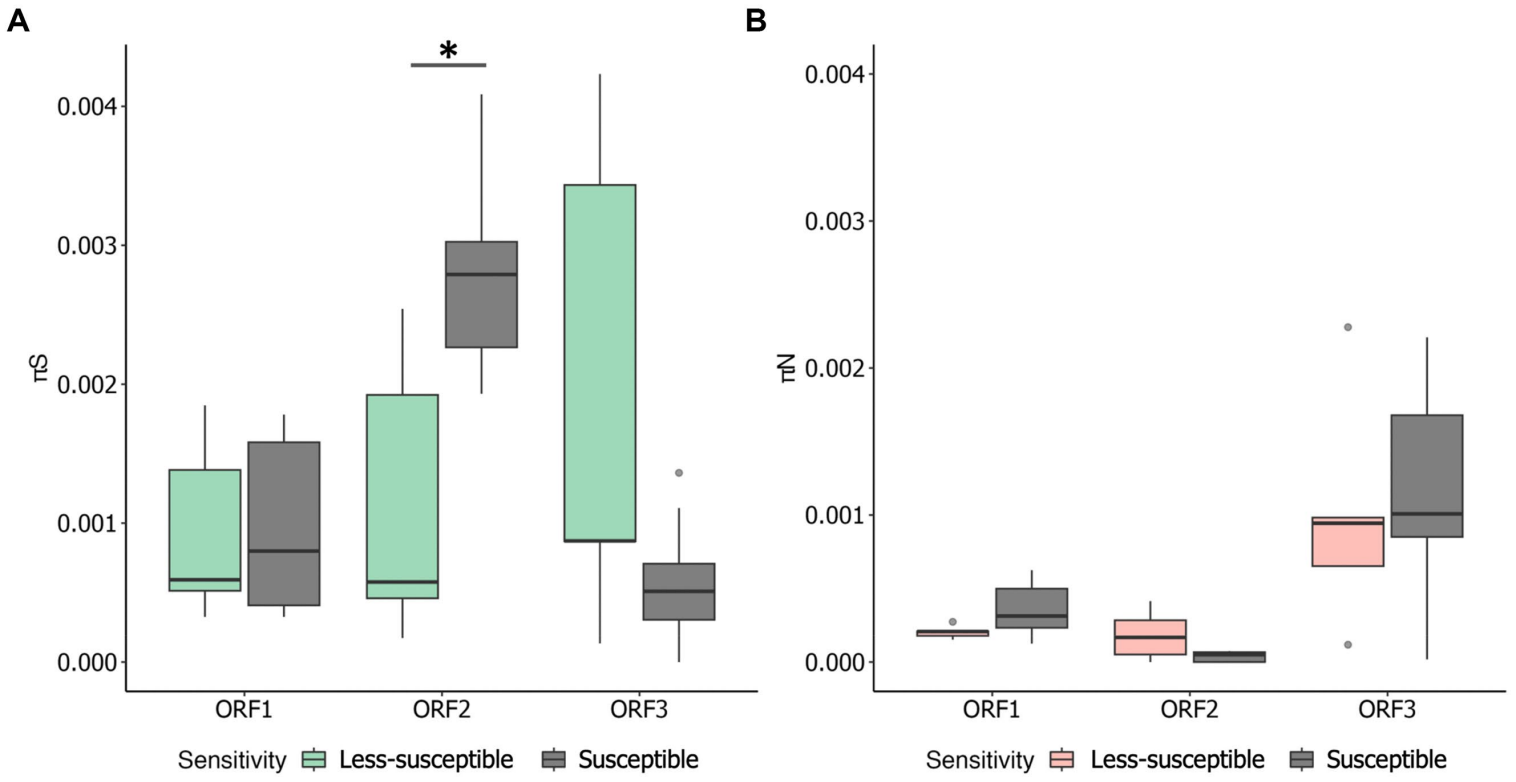
Overall values of synonymous nucleotide diversity (π_S_) **(A)** and nonsynonymous nucleotide diversity (π_N_) **(B)** in each coding region of MNV populations. Statistical differences were calculated using the Mann–Whitney *U* test. An asterisk represents *p* < 0.05.

**Figure 4:**
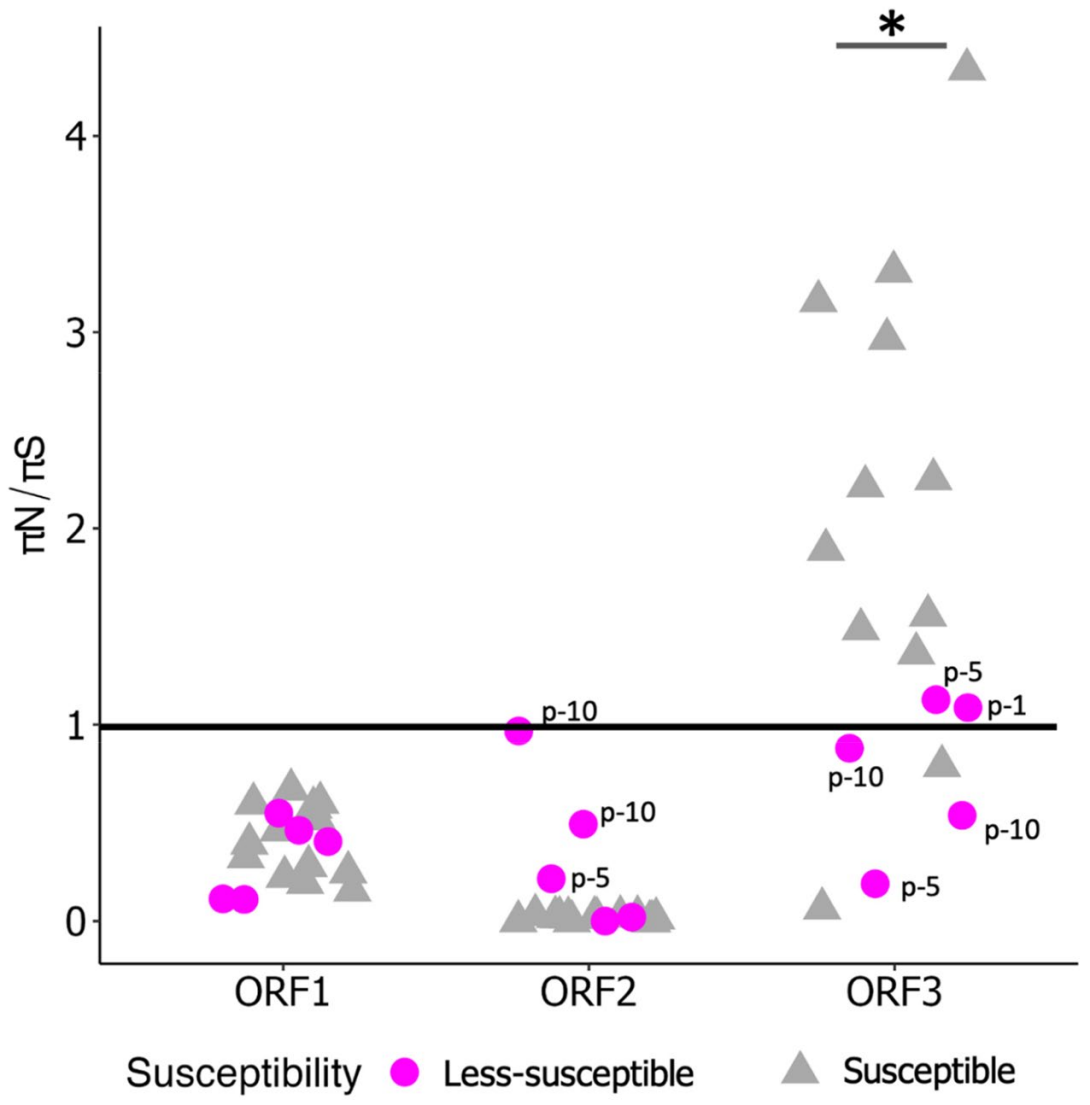
Value of π_N_/π_S_ in each coding region of less-susceptible and susceptible MNV populations. Several less-susceptible populations were shown with labels from the first, fifth, and tenth passages (p-1, p-5, p-10) in ORF2 and ORF3. Statistical differences were calculated using the Mann–Whitney *U* test. An asterisk represents *p* < 0.05.

We examined π_S_ and π_N_ in each ORF (Figures 3A,B). Overall, the mean π_S_ in ORF2, encoding major capsid protein (VP1), of the less-susceptible populations was significantly lower (Mann–Whitney *U* test, *p* = 0.004) than that of the susceptible populations. Furthermore, the mean value of π_N_ of VP1 in the less-susceptible populations was higher than that in the susceptible populations, although the difference was not significant. We also estimated the average value of the π_N_/π_S_ ratio in all coding regions. The average values of π_N_/π_S_ in ORF1 and ORF2 were < 1, and the difference between the less-susceptible and susceptible populations was not significant (*p* = 0.39 and *p* = 0.09, respectively, based on the Mann–Whitney *U* test). Several less-susceptible MNV populations, especially MNV populations from the later passages (fifth and tenth) of both rounds that were exposed to 50 ppm of initial free chlorine, showed significantly higher values of π_N_/π_S_ in ORF2 than the susceptible populations (Mann–Whitney *U* test, *p* = 0.01; Figure 4). The average values of π_N_/π_S_ of ORF3, encoding minor capsid protein (VP2), in the less-susceptible populations were < 1, which was significantly lower than that in susceptible populations (Mann–Whitney *U* test, *p* = 0.02; Figure 4).

**Figure 5:**
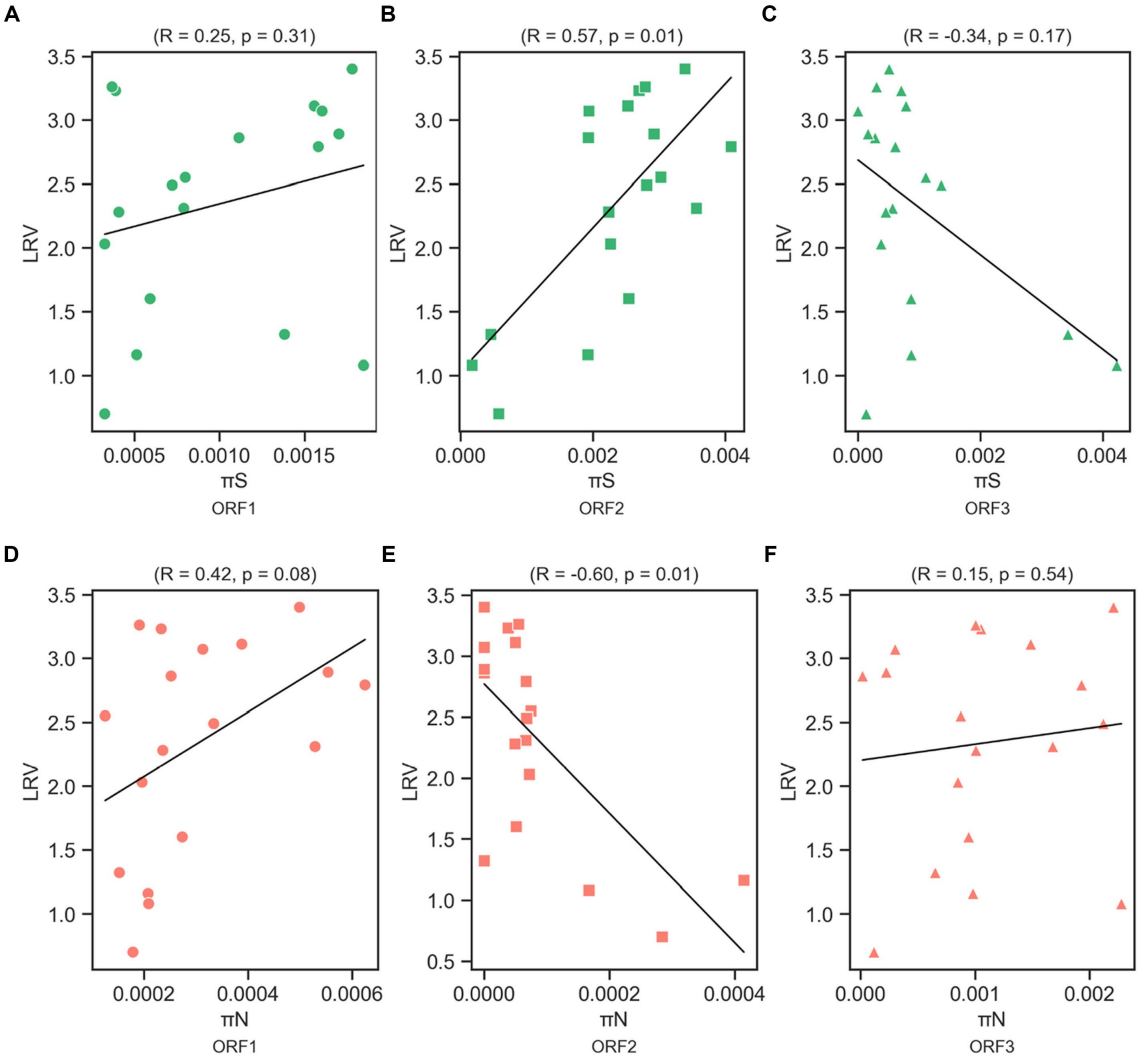
Correlation between log_10_ reduction value (LRV) due to chlorine exposure with synonymous nucleotide diversity (π_S_) in ORF1 **(A)**, ORF2 **(B)**, and ORF3 **(C)**, and nonsynonymous nucleotide diversity (π_N_) in ORF1 **(D)**, ORF2 **(E)**, and ORF3 **(F)** of the MNV genome based on Spearman’s correlation coefficient (R) and *p*-value.

We determined the Spearman’s correlation coefficient (R) to investigate the statistical correlation between chlorine susceptibility in terms of LRV and synonymous and nonsynonymous nucleotide diversity in each coding region (Figure 5). A significant positive correlation was observed between the chlorine susceptibility and π_S_ of ORF2 (R = 0.57, *p* = 0.01; Figure 5B). In contrast, a significant negative correlation was found between chlorine susceptibility and π_N_ in ORF2 (R = −0.60, *p* = 0.01; Figure 5E). The other correlations differed between the coding regions but were not statistically significant. Furthermore, several residual plots for the data sets of π_S_, π_N,_ and LRV showed that the residuals were randomly scattered around the horizontal line at zero, indicating that the variability of the residuals was consistent across different fitted values (Supplementary Figures S2, S3). However, the distribution and characteristics of the data may vary across different ranges of the fitted values. Some residual plots showed random scatter in the higher range of fitted values and only a few residuals in the lower range, which can be influenced by the limited observations.

### 3.3 Frequency of SNPs in MNV populations

**Figure 6:**
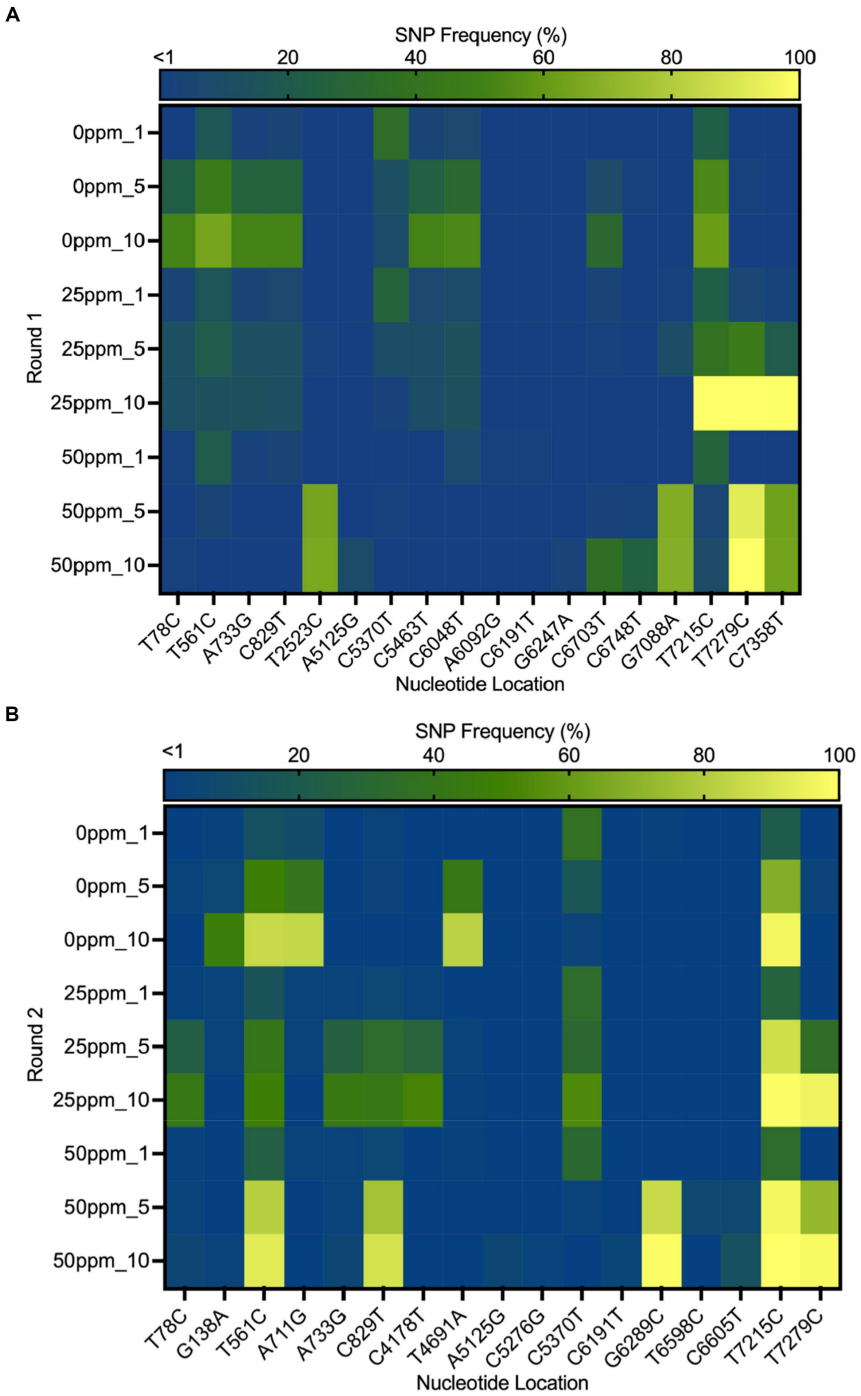
Heat map demonstrating the frequency of the single nucleotide polymorphisms (SNPs) that contained at least 1% of MNV populations in control (0 ppm) treated with initial free chlorine concentrations of 25 and 50 ppm from the first, fifth, and tenth passages in the first **(A)** and second **(B)** rounds. The gradual change in color from dark blue to yellow indicates low to high frequency of mutations in the nucleotide location.

The heat map in Figure 6 shows the distribution of SNPs in the different MNV populations. The nucleotide and amino acid changes in all MNV populations are summarized in Supplementary Table S1. More nonsynonymous mutations were identified in VP1 of the less-susceptible populations than in the susceptible populations at positions 5,125, 5,276, 6,289, 6,598, and 6,605, especially in MNV populations exposed to 50 ppm of initial free chlorine from the fifth and tenth passages. These mutations caused amino acid alterations from threonine to alanine, threonine to serine, leucine to valine, phenylalanine to leucine, and valine to alanine in the major capsid proteins (T24A, T74S, L412V, F515L, and V517A). The frequency of these nonsynonymous mutations ranged from 1 to 85%. The predicted three-dimensional structures of VP1 with nonsynonymous mutations identified in the susceptible and less-susceptible MNV populations is shown in Supplementary Figure S4.

Furthermore, nonsynonymous mutations at nucleotide positions 561, 829, and 7,215 were found in the less-susceptible and susceptible populations in all rounds. These mutations exhibited >90% frequency in a particular passage. Mutations at positions 561 and 829 resulted in amino acid changes from proline to serine and from threonine to methionine (P186S and T275M), respectively, in non-structural proteins. Another mutation at position 7,215 in VP2 resulted in the replacement of tryptophan with arginine (W179R). In addition, all treated populations exposed to chlorine harbored a nonsynonymous mutation at position 7,279, resulting in a phenylalanine to serine substitution in VP2 (F200S) (Supplementary Figure S5). The frequency of this single dominant mutation increased with passage, exceeding 90% by the tenth passage.

### 3.4 Estimation of nucleotide substitution rates

The average nucleotide substitution rates for each ORF shown in Table 1 did not differ significantly among the control and treated populations (Mann–Whitney *U* test, *p* > 0.05). Overall, the nucleotide substitution rate was the lowest in ORF1 of all MNV populations, whereas ORF3 exhibited the highest rate. The nucleotide substitution rates were 10^−5^ substitution/site/passage, ranging from 4.61 (0.001–16.08) to 12.63 (0.13–38.85) in ORF1, 13.32 (0.002–46.88) to 41.60 (0.50–134.39) in ORF2, and 66.71 (0.09–207.09) to 116.50 (2.09–343.58) in ORF3. Furthermore, higher nucleotide substitution rates were observed in ORF3 of the chlorine-treated MNV populations than those in the control. Although the differences were not statistically significant, the results suggest that chlorine exposure might have applied selective pressure on ORF3 of the treated MNV populations. This could potentially result in genetic changes that are more prone to occur in this region.

**Table 1:** Nucleotide substitution rates in three different MNV genomic regions in control and MNV populations exposed to 25 and 50 ppm initial free chlorine.

MNV populations	Nucleotide substitution rate^a^ (95% HPD^b^)		
	ORF1	ORF2	ORF3
Round 1-Control (0 ppm)	8.67 (0.02–26.80)	41.60 (0.5–134.39)	69.74 (0.97–227.32)
Round 2-Control (0 ppm)	11.76 (0.17–36.13)	14.79 (0.005–48.31)	66.71 (0.09–207.09)
Round 1-Treated (25 ppm)	4.61 (0.001–16.08)	13.32 (0.002–46.88)	116.50 (2.09–343.58)
Round 2-Treated (25 ppm)	8.97 (0.06–28.50)	28.91 (0.14–95.83)	106.30 (2.09–324.32)
Round 1-Treated (50 ppm)	8.66 (0.02–26.81)	15.18 (0.003–52.68)	98.14 (1.11–300.78)
Round 2-Treated (50 ppm)	12.63 (0.13–38.85)	26.70 (0.16–88.60)	101.50 (1.21–305.59)

^a^Expressed as 10^-5^ substitutions/site/passage.

^b^95% highest probability density values.

## 4 Discussion

This study investigated the adaptation of MNV populations exposed to chlorine in experimental adaptation to understand the mechanism of development of the less-susceptible virus populations. We also performed whole-genome sequencing to obtain insights regarding the genetic characteristics of the less-susceptible MNV populations. The chlorine sensitivity of the MNV populations varied with the initial free chlorine concentration. The five MNV populations exposed to 50 ppm free chlorine (injection rate) displayed lower median values of LRV than the control populations, suggesting reduced susceptibility to chlorine (Figure 2). Nucleotide diversity analysis revealed differences in π_S_ and π_N_ of the less-susceptible populations, particularly in VP1. The less-susceptible populations displayed significantly low π_S_, and three of the populations showed an increase in π_N_/π_S_ at the fifth and tenth passage in VP1 (Figures 3, 4). Spearman’s correlation analysis showed a significant positive correlation between chlorine susceptibility and π_S_, whereas a significant negative correlation was observed between chlorine susceptibility and π_N_ in VP1 (Figure 5). Over 90% of all chlorine-treated MNV populations harbored a dominant mutation in VP2 (Figure 6). Moreover, the chlorine-treated populations exhibited higher nucleotide substitution rate in VP2 than in the control populations (Table 1). These findings suggested a link between susceptibility to chlorine disinfection and nucleotide diversity in the MNV populations.

Several mechanisms via which virus populations adapt to disinfection have been reported. The responses of virus populations, including rotavirus, echovirus 11, coxsackievirus B5, MS2 coliphage, and MNV to exposure to various disinfectants, such as chlorine dioxide, ultraviolet radiation, calcium hydroxide, and ozone, have been investigated (Carratalà et al., 2017; Zhong et al., 2017a,b; Torii et al., 2021; Kadoya et al., 2022; Oishi et al., 2022). A study found that MNV populations exhibited lower susceptibility to calcium hydroxide due to a specific mutation in the VP1 protein, which causes the substitution of lysine at position 345 with arginine (Oishi et al., 2022). In the present study, a highly frequent nonsynonymous mutation was identified at position 7,279 of VP2 in >90% MNV populations exposed to initial free chlorine concentrations of 25 and 50 ppm. VP2 is involved in viral entry and modulation of the immune response (Campillay-Véliz et al., 2020; Liao et al., 2022), and is a versatile structural protein with the highest nucleotide substitution rate among other norovirus proteins (Mauroy et al., 2017; Campillay-Véliz et al., 2020). Rachmadi et al. also identified a change from phenylalanine to serine at site 200 on VP2 in the MNV populations exposed to chlorine (Rachmadi et al., 2018). However, they only sequenced the capsid proteins, VP1 and VP2, referring to different reference sequences in the bioinformatics analysis, leading to a discrepancy of one nucleotide base at the mutation location (Rachmadi et al., 2018). As this mutation was also detected in the susceptible MNV populations in this study, it may not be the only cause of the reduced susceptibility to chlorine. The results of whole-genome sequencing suggested that the identified single dominant mutation in VP2 does not alter chlorine susceptibility in MNV populations.

Based on synonymous and nonsynonymous nucleotide diversities, particularly in VP1, we concluded that the adaptation mechanism of the susceptible and less-susceptible populations of MNV were distinct. Notably, the susceptible populations had significantly higher π_S_ in VP1 than other proteins (Figure 3A). These results suggest that the appearance of synonymous mutations could be caused by weak selective pressures (de Jong et al., 2024). Meanwhile, the less-susceptible populations had significantly lower π_S_ in VP1 than the susceptible populations (Figure 3A). This can be explained by the diverse secondary structure of the genomic RNA, which alters the interactions between nucleotides (Qiao et al., 2022) and influences the charge distribution and overall conformation of the RNA molecules (Roundtree et al., 2017). Norovirus capsids harbor negative surface charge in neutral or basic pH (Kang and Cannon, 2015). A reduction in the negative charge or change in its distribution because of modifications in the genomic RNA reduces the electrostatic repulsive forces between viral particles, facilitating viral aggregation (Mertens and Velev, 2015). Computational methods using the Poisson-Boltzmann equation can be used to calculate the electrostatic potential and identify charge distribution in particular proteins (Zhang et al., 2022). However, further research is required to clarify the relationships between synonymous mutations in the genomic RNA, changes in virion surface charges, and the formation of aggregates.

Several less-susceptible populations from the later passages (fifth and tenth) of both rounds showed significantly higher value of π_N_/π_S_ in ORF2 than other populations (Figure 4). Furthermore, significant negative correlation was observed between chlorine susceptibility and π_N_ in ORF2 (Figure 5E), indicating relaxation of purifying selection during extended chlorine exposure. Therefore, increased genetic diversity at nonsynonymous sites in VP1 is associated with viral adaptation to chlorine. This finding is consistent with that of Kadoya et al., in which populations of rotaviruses less susceptible to chlorine had higher nonsynonymous diversity in the capsid-coding region, and the formation of aggregates among virions with diverse microstructures and surface charges was proposed as a plausible explanation (Kadoya et al., 2022). Major and minor virus mutants may aggregate into clusters, creating a collective shield that results in the viruses within these clusters being less exposed to chlorine (Kadoya et al., 2022). The study further showed that virus clusters may contribute to increasing genetic diversity within a population and stabilize population size, leading to a reduction in the effectiveness of disinfection, as performed by computational evolutionary simulations (Kadoya et al., 2022). Furthermore, several studies have identified specific mutations related to viral aggregation. A study showed that a mutation in the hemagglutinin-neuraminidase gene of the mumps virus can change the electrostatic surface potential, which influences aggregation (Santos-López et al., 2009). Another study also observed that a mutation in the capsid protein of the Sindbis virus can affect aggregation (Ferreira et al., 2003). Detailed study of viral aggregates, for instance, using electron microscopy, nanoparticle tracking analysis, flow virometry, or tunable resistive pulse sensing, can be beneficial for characterizing viral aggregation in terms of size distribution, morphology, surface charge, composition, structure, and stability (Heider and Metzner, 2014; Zamora and Aguilar, 2018; Carvalho et al., 2022).

Besides nucleotide diversity, the emergence of certain nonsynonymous mutations can also affect chlorine susceptibility by influencing the efficiency of viral replication. Amino acid substitutions in VP1 can potentially impact critical functions of viral capsids, including protecting the viral genome and facilitating interactions between the virus and host cells (Campillay-Véliz et al., 2020). ORF2 encoding VP1 consists of a well-conserved shell (S) domain, the interior surface of the virus capsid surrounding the RNA, and a protruding (P) domain (Nelson et al., 2018). The P domain is divided into the proximal and distal subdomains, P1 and P2, respectively (Supplementary Figure S6; Zhu et al., 2016; Nelson et al., 2018). The P1 subdomain consists of a single *α* helix and eight *β* strands, while the P2 subdomain appears as an insertion in P1 that forms a six-stranded antiparallel *β*-barrel (Katpally et al., 2010). We detected amino acid substitutions, F515L and V517A, within the *β* strand of the P1 subdomain, and L412V within the P2 subdomain, by mapping the nucleotide changes in the P domain of MNV VP1 in complex with its cellular receptor, CD300lf and visualizing using the UCSF ChimeraX version 1.7.1 (Supplementary Figure S7; Goddard et al., 2018; Nelson et al., 2018; Pettersen et al., 2021; Meng et al., 2023). Based on the protein model, we have identified the location of mutations in the P1 and P2 subdomains. However, these mutations are not precisely situated within the receptor binding site (Kilic et al., 2018). Another explanation is that the mutations may impact inter-capsid interactions, regulating the size and stability of the viral capsid by establishing intermolecular contacts between dimeric VP1 subunits (Bertolotti-Ciarlet et al., 2002). Recent studies have also revealed that a single mutation in the P domain of norovirus VP1 significantly improved cellular attachment of the virus during infection (Mills et al., 2023). Further investigation into the functional consequences of these mutations on receptor binding and inter-capsid interactions is necessary to better comprehend their role in determining the susceptibility of virus populations to chlorine.

The use of MNV as a surrogate for human norovirus is one of the limitations of this study. Viral surrogates are used for studying human norovirus owing to the challenges in culturing human norovirus in cell culture systems (Duizer et al., 2004). Although viral surrogates have dissimilar viral receptor characteristics, the genome of MNV is similar and to that of the human norovirus, with which it shares taxonomic proximity (Wobus et al., 2006; Bae and Schwab, 2008; Belliot et al., 2008). Moreover, their properties, including capsid structure and routes of fecal–oral transmission, are similar (Karst et al., 2014; Graziano et al., 2019; Kilic et al., 2019). Therefore, the results of this study using MNV can be extended to human noroviruses (Dimitrov, 2004; Le Pendu et al., 2016; Graziano et al., 2020).

Overall, analysis of the genetic structure of viral populations provided valuable insights regarding the mechanism underlying adaptation to disinfection. Notably, genetic alterations in VP1 occurred due to exposure to a particular concentration of chlorine, which affected the susceptibility of the virus. Our findings provide new insights regarding the application of adequate chlorine for disinfection, which can exert different selection pressures on virus populations, leading to the emergence of less-susceptible populations. One solution to this problem involves the use of multiple types of disinfectants during wastewater treatment (Kadoya et al., 2021). Furthermore, the results suggested that genetic diversity can act as an index or indicator of chlorine susceptibility in MNV populations. Further research on the adaptation of virus populations with different levels of genetic diversity to disinfectants will be useful for understanding the importance of genetic diversity in identifying adaptation mechanisms. In addition, implementation of reverse genetic approaches may be beneficial; the coding regions of wild type MNV can be replaced by the corresponding VP1 sequences of the less-susceptible populations for confirming the coding region responsible for influencing viral sensitivity to chlorine.

## Data availability statement

The datasets presented in this study can be found in online repositories. The names of the repository/repositories and accession number(s) can be found at: https://www.ncbi.nlm.nih.gov/genbank/, SUB12923106 (OQ552767–OQ552784).

## Ethics statement

Ethical approval was not required for the studies on animals in accordance with the local legislation and institutional requirements because only commercially available established cell lines were used.

## Author contributions

AW: Formal analysis, Visualization, Writing – original draft, Writing – review & editing. WO: Conceptualization, Investigation, Methodology, Supervision, Writing – review & editing. AR: Conceptualization, Investigation, Methodology, Writing – review & editing. KK: Supervision, Writing – review & editing. DS: Conceptualization, Funding acquisition, Supervision, Writing – review & editing.

## References

[ref1] Ahmed S. M. Hall A. J. Robinson A. E. Verhoef L. Premkumar P. Parashar U. D.. (2014). Global prevalence of norovirus in cases of gastroenteritis: a systematic review and meta-analysis. Lancet Infect. Dis. 14, 725–730. doi: 10.1016/S1473-3099(14)70767-4, PMID: 24981041 PMC8006533

[ref2] Bae J. Schwab K. J. (2008). Evaluation of murine norovirus, feline Calicivirus, poliovirus, and MS2 as surrogates for human norovirus in a model of viral persistence in surface water and groundwater. Appl. Environ. Microbiol. 74, 477–484. doi: 10.1128/AEM.02095-06, PMID: 18065626 PMC2223264

[ref3] Barron E. L. Sosnovtsev S. V. Bok K. Prikhodko V. Sandoval-Jaime C. Rhodes C. R.. (2011). Diversity of murine norovirus strains isolated from asymptomatic mice of different genetic backgrounds within a single U.S. research institute. PLoS One 6:e21435. doi: 10.1371/journal.pone.0021435, PMID: 21738664 PMC3125191

[ref4] Belliot G. Lavaux A. Souihel D. Agnello D. Pothier P. (2008). Use of murine norovirus as a surrogate to evaluate resistance of human norovirus to disinfectants. Appl. Environ. Microbiol. 74, 3315–3318. doi: 10.1128/AEM.02148-07, PMID: 18378650 PMC2394958

[ref5] Bertolotti-Ciarlet A. White L. J. Chen R. Prasad B. V. V. Estes M. K. (2002). Structural requirements for the assembly of Norwalk virus-like particles. J. Virol. 76, 4044–4055. doi: 10.1128/jvi.76.8.4044-4055.2002, PMID: 11907243 PMC136079

[ref6] Black S. Thurston J. A. Gerba C. P. (2009). Determination of Ct values for chlorine of resistant enteroviruses. J. Environ. Sci. Health A Tox. Hazard. Subst. Environ. Eng. 44, 336–339. doi: 10.1080/10934520802659653, PMID: 19184699

[ref7] Bouckaert R. Vaughan T. G. Barido-Sottani J. Duchêne S. Fourment M. Gavryushkina A.. (2019). BEAST 2.5: an advanced software platform for Bayesian evolutionary analysis. PLoS Comput. Biol. 15:e1006650. doi: 10.1371/journal.pcbi.1006650, PMID: 30958812 PMC6472827

[ref8] Campillay-Véliz C. P. Carvajal J. J. Avellaneda A. M. Escobar D. Covián C. Kalergis A. M.. (2020). Human norovirus proteins: implications in the replicative cycle, pathogenesis, and the host immune response. Front. Immunol. 11:961. doi: 10.3389/fimmu.2020.00961, PMID: 32612600 PMC7308418

[ref9] Carratalà A. Shim H. Zhong Q. Bachmann V. Jensen J. D. Kohn T. (2017). Experimental adaptation of human echovirus 11 to ultraviolet radiation leads to resistance to disinfection and ribavirin. Virus Evol 3:35. doi: 10.1093/ve/vex035, PMID: 29225923 PMC5714166

[ref10] Carstens E. B. (2010). Ratification vote on taxonomic proposals to the international committee on taxonomy of viruses (2009). Arch. Virol. 155, 133–146. doi: 10.1007/s00705-009-0547-x, PMID: 19960211 PMC7086975

[ref11] Carvalho S. B. Silva R. J. S. Sousa M. F. Q. Peixoto C. Roldão A. Carrondo M. J. T.. (2022). Bioanalytics for influenza virus-like particle characterization and process monitoring. Front. Bioeng. Biotechnol. 10:805176. doi: 10.3389/fbioe.2022.805176, PMID: 35252128 PMC8894879

[ref12] Chhabra P. de Graaf M. Parra G. I. Chan M. C. W. Green K. Martella V.. (2019). Updated classification of norovirus Genogroups and genotypes. J. Gen. Virol. 100, 1393–1406. doi: 10.1099/JGV.0.001318, PMID: 31483239 PMC7011714

[ref13] Collivignarelli M. C. Abbà A. Benigna I. Sorlini S. Torretta V. (2018). Overview of the Main disinfection processes for wastewater and drinking water treatment plants. Sustainability (Switzerland) 10:86. doi: 10.3390/su10010086

[ref14] Cromeans T. L. Kahler A. M. Hill V. R. (2010). Inactivation of adenoviruses, enteroviruses, and murine norovirus in water by free chlorine and Monochloramine. Appl. Environ. Microbiol. 76, 1028–1033. doi: 10.1128/AEM.01342-09, PMID: 20023080 PMC2820971

[ref15] Danecek P. Bonfield J. K. Liddle J. Marshall J. Ohan V. Pollard M. O.. (2021). Twelve years of SAMtools and BCFtools. Gigascience 10:giab008. doi: 10.1093/gigascience/giab008, PMID: 33590861 PMC7931819

[ref16] de Jong M. J. van Oosterhout C. Hoelzel A. R. Janke A. (2024). Moderating the neutralist–Selectionist debate: exactly which propositions are we debating, and which arguments are valid? Biol. Rev. 99:13010. doi: 10.1111/brv.13010 37621151

[ref17] Debbink K. Lindesmith L. C. Baric R. S. (2014). The state of norovirus vaccines. Clin. Infect. Dis. 58, 1746–1752. doi: 10.1093/cid/ciu120, PMID: 24585561 PMC4036685

[ref18] Dias E. Ebdon J. Taylor H. (2019). Estimating the concentration of viral pathogens and Indicator organisms in the final effluent of wastewater treatment processes using stochastic modelling. Microb Risk Anal 11, 47–56. doi: 10.1016/j.mran.2018.08.003

[ref19] Dimitrov D. S. (2004). Virus entry: molecular mechanisms and biomedical applications. Nat. Rev. Microbiol. 2, 109–122. doi: 10.1038/nrmicro817, PMID: 15043007 PMC7097642

[ref20] Duizer E. Koopmans M. (2006). Tracking emerging pathogens: the case of noroviruses. Emerg Foodborne Pathogens, 77–110. doi: 10.1533/9781845691394.1.77

[ref21] Duizer E. Schwab K. J. Neill F. H. Atmar R. L. Koopmans M. P. G. Estes M. K. (2004). Laboratory efforts to cultivate noroviruses. J. Gen. Virol. 85, 79–87. doi: 10.1099/vir.0.19478-0, PMID: 14718622

[ref22] Ferreira D. Hernandez R. Horton M. Brown D. T. (2003). Morphological variants of Sindbis virus produced by a mutation in the capsid protein. Virology 307, 54–66. doi: 10.1016/S0042-6822(02)00034-X, PMID: 12667814

[ref23] Goddard T. D. Huang C. C. Meng E. C. Pettersen E. F. Couch G. S. Morris J. H.. (2018). UCSF ChimeraX: meeting modern challenges in visualization and analysis. Protein Sci. 27, 14–25. doi: 10.1002/pro.3235, PMID: 28710774 PMC5734306

[ref24] Gonzalez-Hernandez M. B. Cunha J. B. Wobus C. E. (2012). Plaque assay for murine norovirus. J. Vis. Exp. 1–6:e4297. doi: 10.3791/4297, PMID: 22951568 PMC3487293

[ref25] Graziano V. R. Walker F. C. Kennedy E. A. Wei J. Ettayebi K. Strine M. S.. (2020). CD300lf is the primary physiologic receptor of murine norovirus but not human norovirus. PLoS Pathog. 16:e1008242. doi: 10.1371/journal.ppat.1008242, PMID: 32251490 PMC7162533

[ref26] Graziano V. R. Wei J. Wilen C. B. (2019). Norovirus attachment and entry. Viruses 11:495. doi: 10.3390/v11060495, PMID: 31151248 PMC6630345

[ref27] Heider S. Metzner C. (2014). Quantitative real-time single particle analysis of Virions. Virology 462-463, 199–206. doi: 10.1016/j.virol.2014.06.005, PMID: 24999044 PMC4139191

[ref28] Hellmér M. Paxéus N. Magnius L. Enache L. Arnholm B. Johansson A.. (2014). Detection of pathogenic viruses in sewage provided early warnings of hepatitis a virus and norovirus outbreaks. Appl. Environ. Microbiol. 80, 6771–6781. doi: 10.1128/AEM.01981-14, PMID: 25172863 PMC4249052

[ref29] Hierholzer J. C. Killington R. A. (1996). Virology methods manual. Cambridge, MA: Academic Press.

[ref30] Jumper J. Evans R. Pritzel A. Green T. Figurnov M. Ronneberger O.. (2021). Highly accurate protein structure prediction with AlphaFold. Nature 596, 583–589. doi: 10.1038/s41586-021-03819-2, PMID: 34265844 PMC8371605

[ref31] Kadoya S. Katayama H. Sano D. (2021). Virus disinfection and population genetics: toward the control of waterborne virus diseases by water engineering. Curr. Pollut. Rep. 7, 407–416. doi: 10.1007/s40726-021-00189-1/Published

[ref32] Kadoya S. Urayama S. Nunoura T. Hirai M. Takaki Y. Kitajima M.. (2022). The Intrapopulation genetic diversity of RNA virus may influence the sensitivity of chlorine disinfection. Front. Microbiol. 13:839513. doi: 10.3389/fmicb.2022.839513, PMID: 35668760 PMC9163991

[ref33] Kahler A. M. Cromeans T. L. Roberts J. M. Hill V. R. (2010). Effects of source water quality on chlorine inactivation of adenovirus, Coxsackievirus, echovirus, and murine norovirus. Appl. Environ. Microbiol. 76, 5159–5164. doi: 10.1128/AEM.00869-10, PMID: 20562285 PMC2916456

[ref34] Kang W. Cannon J. L. (2015). A membrane-based electro-separation method (MBES) for sample clean-up and norovirus concentration. PLoS One 10:e0141484. doi: 10.1371/journal.pone.0141484, PMID: 26513464 PMC4625962

[ref35] Karst S. M. Wobus C. E. Goodfellow I. G. Green K. Y. Virgin H. W. (2014). Advances in norovirus biology. Cell Host Microbe 15, 668–680. doi: 10.1016/j.chom.2014.05.015, PMID: 24922570 PMC4113907

[ref36] Katpally U. Voss N. R. Cavazza T. Taube S. Rubin J. R. Young V. L.. (2010). High-resolution Cryo-Electron microscopy structures of murine norovirus 1 and rabbit hemorrhagic disease virus reveal marked flexibility in the receptor binding domains. J. Virol. 84, 5836–5841. doi: 10.1128/jvi.00314-10, PMID: 20335264 PMC2876586

[ref37] Kilic T. Koromyslova A. Hansman G. S. (2019). Structural basis for human norovirus capsid binding to bile acids. J. Virol. 93:e01581. doi: 10.1128/jvi.01581-18, PMID: 30355683 PMC6321941

[ref38] Kilic T. Koromyslova A. Malak V. Hansman G. S. (2018). Atomic structure of the murine norovirus protruding domain and soluble CD300lf receptor complex. J. Virol. 92:e00413. doi: 10.1128/jvi.00413-18, PMID: 29563286 PMC5952153

[ref39] Le Pendu J. Rydell G. E. Nasir W. Larson G. (2016). “Human Norovirus Receptors,” in Viral Gastroenteritis. eds. Svensson L. Desselberger U. Greenberg H. Estes M. (Elsevier), 379–396.

[ref40] Liao Y. Wang L. Hong X. Gao J. Zuo Y. Liang Y.. (2022). The VP2 protein exhibits cross-interaction to the VP1 protein in norovirus GII.17. Infect. Genet. Evol. 100:105265. doi: 10.1016/j.meegid.2022.105265 35272046

[ref41] Lin Q. Lim J. Y. C. Xue K. Yew P. Y. M. Owh C. Chee P. L.. (2020). Sanitizing agents for virus inactivation and disinfection. VIEW 1:e16. doi: 10.1002/viw2.16, PMID: 34766164 PMC7267133

[ref42] Lopman B. A. Steele D. Kirkwood C. D. Parashar U. D. (2016). The vast and varied global burden of norovirus: prospects for prevention and control. PLoS Med. 13:e1001999. doi: 10.1371/journal.pmed.1001999, PMID: 27115709 PMC4846155

[ref43] Lysén M. Thorhagen M. Brytting M. Hjertqvist M. Andersson Y. Hedlund K. O. (2009). Genetic diversity among food-borne and waterborne norovirus strains causing outbreaks in Sweden. J. Clin. Microbiol. 47, 2411–2418. doi: 10.1128/JCM.02168-08, PMID: 19494060 PMC2725682

[ref44] Marks P. J. Vipond I. D. Carlisle D. Deakin D. Fey R. E. Caul E. O. (2000). Evidence for airborne transmission of Norwalk-like virus (NLV) in a hotel restaurant. Epidemiol. Infect. 124, 481–487. doi: 10.1017/S0950268899003805, PMID: 10982072 PMC2810934

[ref45] Mauroy A. Taminiau B. Nezer C. Ghurburrun E. Baurain D. Daube G.. (2017). High-throughput sequencing analysis reveals the genetic diversity of different regions of the murine norovirus genome during *in vitro* replication. Arch. Virol. 162, 1019–1023. doi: 10.1007/s00705-016-3179-y, PMID: 27942973

[ref46] McFadden N. Bailey D. Carrara G. Benson A. Chaudhry Y. Shortland A.. (2011). Norovirus regulation of the innate immune response and apoptosis occurs via the product of the alternative open Reading frame 4. PLoS Pathog. 7:e1002413. doi: 10.1371/journal.ppat.1002413, PMID: 22174679 PMC3234229

[ref47] Meng E. C. Goddard T. D. Pettersen E. F. Couch G. S. Pearson Z. J. Morris J. H.. (2023). UCSF ChimeraX: tools for structure building and analysis. Protein Sci. 32:e4792. doi: 10.1002/pro.4792, PMID: 37774136 PMC10588335

[ref48] Mertens B. S. Velev O. D. (2015). Characterization and control of surfactant-mediated norovirus interactions. Soft Matter 11, 8621–8631. doi: 10.1039/c5sm01778e, PMID: 26378627 PMC4666303

[ref49] Mills J. T. Minogue S. C. Snowden J. S. Arden W. K. C. Rowlands D. J. Stonehouse N. J.. (2023). Amino acid substitutions in norovirus VP1 dictate host dissemination via variations in cellular attachment. J. Virol. 97:e0171923. doi: 10.1128/jvi.01719-23, PMID: 38032199 PMC10734460

[ref9002] Nei M. Gojobori T. (1986). Simple methods for estimating the numbers of synonymous and nonsynonymous nucleotide substitutions. Mol. Biol. Evol. 3. doi: 10.1093/oxfordjournals.molbev.a040410 3444411

[ref50] Nelson C. W. Hughes A. L. (2015). Within-host nucleotide diversity of virus populations: insights from next-generation sequencing. Infect. Genet. Evol. 30, 1–7. doi: 10.1016/j.meegid.2014.11.026, PMID: 25481279 PMC4316684

[ref51] Nelson C. W. Moncla L. H. Hughes A. L. (2015). SNPGenie: estimating evolutionary parameters to detect natural selection using pooled next-generation sequencing data. Bioinformatics 31, 3709–3711. doi: 10.1093/bioinformatics/btv449, PMID: 26227143 PMC4757956

[ref52] Nelson C. A. Wilen C. B. Dai Y. N. Orchard R. C. Kim A. S. Stegeman R. A.. (2018). Structural basis for murine norovirus engagement of bile acids and the CD300lf receptor. Proc. Natl. Acad. Sci. USA 115, E9201–E9210. doi: 10.1073/pnas.1805797115, PMID: 30194229 PMC6166816

[ref53] Oishi W. Sato M. Kubota K. Ishiyama R. Takai-Todaka R. Haga K.. (2022). Experimental adaptation of murine norovirus to calcium hydroxide. Front. Microbiol. 13:848439. doi: 10.3389/fmicb.2022.848439, PMID: 35432235 PMC9009222

[ref54] Pan K. Deem M. W. (2011). Quantifying selection and diversity in viruses by entropy methods, with application to the Haemagglutinin of H3N2 influenza. J. R. Soc. Interface 8, 1644–1653. doi: 10.1098/rsif.2011.0105, PMID: 21543352 PMC3177615

[ref55] Pettersen E. F. Goddard T. D. Huang C. C. Meng E. C. Couch G. S. Croll T. I.. (2021). UCSF ChimeraX: structure visualization for researchers, educators, and developers. Protein Sci. 30, 70–82. doi: 10.1002/pro.3943, PMID: 32881101 PMC7737788

[ref56] Qiao Z. Ye Y. Szczuka A. Harrison K. R. Dodd M. C. Wigginton K. R. (2022). Reactivity of viral nucleic acids with chlorine and the impact of virus Encapsidation. Environ. Sci. Technol. 56, 218–227. doi: 10.1021/acs.est.1c04239, PMID: 34905340

[ref57] Rachmadi A. T. Kitajima M. Watanabe K. Yaegashi S. Serrana J. Nakamura A.. (2018). Free-chlorine disinfection as a selection pressure on norovirus. Appl. Environ. Microbiol. 84, 1–14. doi: 10.1128/AEM PMC6007107 29703740

[ref58] Raghwani J. Pybus O. G. Illingworth C. J. R. (2016). “Population genetic modeling of viruses” in Virus evolution: Current research and future directions (Poole: Caister Academic Press), 293–327.

[ref59] Rambaut A. Drummond A. J. Xie D. Baele G. Suchard M. A. (2018). Posterior summarization in Bayesian Phylogenetics using Tracer 1.7. Syst. Biol. 67, 901–904. doi: 10.1093/sysbio/syy032, PMID: 29718447 PMC6101584

[ref60] Roundtree I. A. Evans M. E. Pan T. He C. (2017). Dynamic RNA modifications in gene expression regulation. Cell 169, 1187–1200. doi: 10.1016/j.cell.2017.05.045, PMID: 28622506 PMC5657247

[ref61] Sanjuán R. (2008). “Quasispecies” in Encyclopedia of virology (Amsterdam: Elsevier Ltd), 359–365.

[ref62] Santos-López G. Scior T. Del Tránsito Borraz-Argüello M. Vallejo-Ruiz V. Herrera-Camacho I. Tapia-Ramírez J.. (2009). Structure-function analysis of two variants of mumps virus hemagglutinin-neuraminidase protein. Braz. J. Infect. Dis. 13, 24–34. doi: 10.1590/S1413-86702009000100007, PMID: 19578626

[ref63] Tajima F. (1989). Statistical method for testing the neutral mutation hypothesis by DNA polymorphism. Genetics 123, 585–595. doi: 10.1093/genetics/123.3.585, PMID: 2513255 PMC1203831

[ref64] Torii S. Miura F. Itamochi M. Haga K. Katayama K. Katayama H. (2021). Impact of the heterogeneity in free chlorine, UV254, and ozone susceptibilities among Coxsackievirus B5 on the prediction of the overall inactivation efficiency. Environ. Sci. Technol. 55, 3156–3164. doi: 10.1021/acs.est.0c07796, PMID: 33583178

[ref65] USEPA (2020). Disinfection profiling and benchmarking: Technical guidance manual. USEPA: Washington, DC.

[ref66] Verhoef L. Hewitt J. Barclay L. Ahmed S. M. Lake R. Hall A. J.. (2015). Norovirus genotype profiles associated with foodborne transmission, 1999–2012. Emerg. Infect. Dis. 21, 592–599. doi: 10.3201/eid2104.141073, PMID: 25811368 PMC4378480

[ref67] Weinberg G. A. (2019). Outbreak epidemiology: one of many new Frontiers of norovirus biology. J. Infect. Dis. 219, 1349–1352. doi: 10.1093/infdis/jiy570, PMID: 30445582

[ref68] Wobus C. E. Thackray L. B. Virgin H. W. (2006). Murine norovirus: a model system to study norovirus biology and pathogenesis. J. Virol. 80, 5104–5112. doi: 10.1128/jvi.02346-05, PMID: 16698991 PMC1472167

[ref69] Zamora J. L. R. Aguilar H. C. (2018). Flow Virometry as a tool to study viruses. Methods 134-135, 87–97. doi: 10.1016/j.ymeth.2017.12.011, PMID: 29258922 PMC5815898

[ref70] Zhang Z. Zhang J. Wang J. (2022). Surface charge changes in spike RBD mutations of SARS-CoV-2 and its variant strains Alter the virus evasiveness via HSPGs: a review and mechanistic hypothesis. Front. Public Health 10:952916. doi: 10.3389/fpubh.2022.952916, PMID: 36091499 PMC9449321

[ref71] Zhong Q. Carratalà A. Ossola R. Bachmann V. Kohn T. (2017a). Cross-resistance of UV- or chlorine dioxide-resistant echovirus 11 to other disinfectants. Front. Microbiol. 8:1928. doi: 10.3389/fmicb.2017.01928, PMID: 29046672 PMC5632658

[ref72] Zhong Q. Carratalà A. Shim H. Bachmann V. Jensen J. D. Kohn T. (2017b). Resistance of echovirus 11 to ClO2 is associated with enhanced host receptor use, altered entry routes, and high fitness. Environ. Sci. Technol. 51, 10746–10755. doi: 10.1021/acs.est.7b03288, PMID: 28837336 PMC5607461

[ref73] Zhu S. Watanabe M. Kirkpatrick E. Murray A. B. Sok R. Karst S. M. (2016). Regulation of norovirus virulence by the VP1 protruding domain correlates with B cell infection efficiency. J. Virol. 90, 2858–2867. doi: 10.1128/jvi.02880-15, PMID: 26719276 PMC4810633

