# Supplementary material for: Genetic diversity of murine norovirus populations less susceptible to chlorine
Source: Front Microbiol. 2024 Apr 30;15:1372641. doi: 10.3389/fmicb.2024.1372641 (PMC11091384; doi:10.3389/fmicb.2024.1372641)
Supplement: Supplementary file 1 [file Data_Sheet_1.pdf]

Supplementary Material

1 Supplementary Figures and Tables

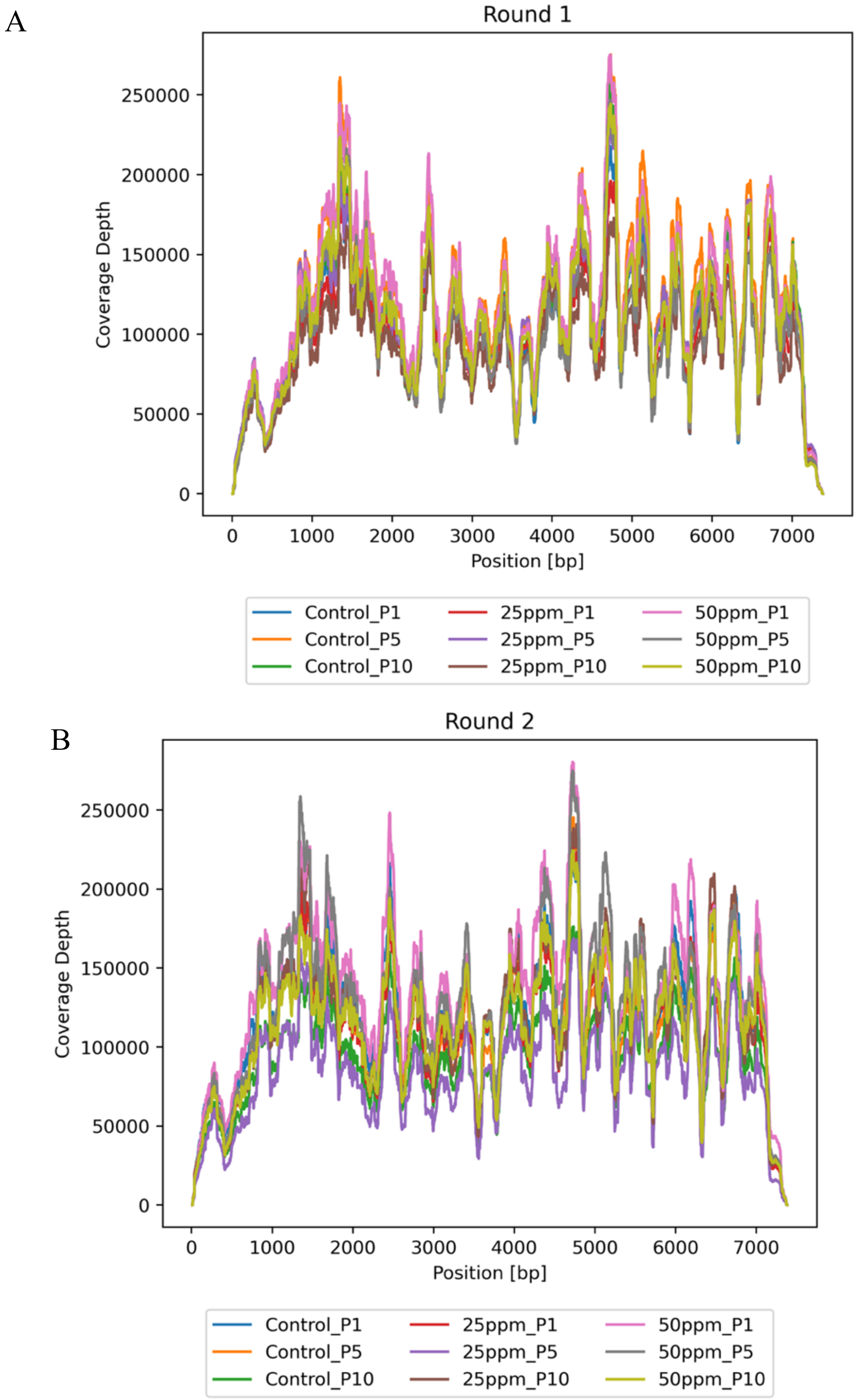

Supplementary Figure 1. Depth of Sequencing Coverage of entire genome sequence in control and treated MNV populations (25 ppm and 50 ppm) in passage 1 (P1), passage 5 (P5), and passage 10 (P10) of round 1 **(A)** and round 2 **(B)**

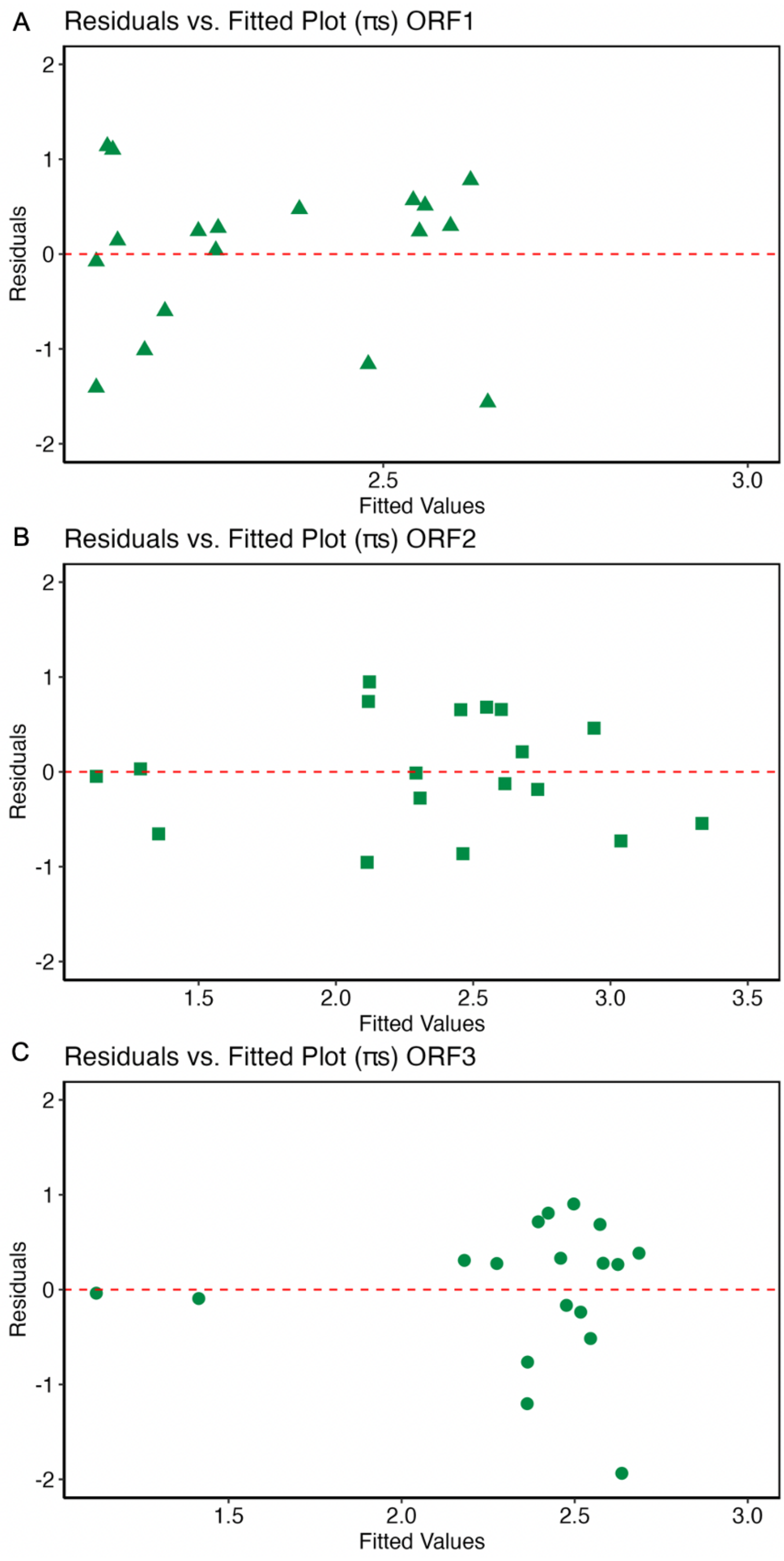

Supplementary Figure 2. The residuals versus fits plot for the data sets of synonymous nucleotide diversity (π_S) and Log_{10} Reduction Value (LRV) in ORF1 **(A)**, ORF2 **(B)**, and ORF3 **(C)**

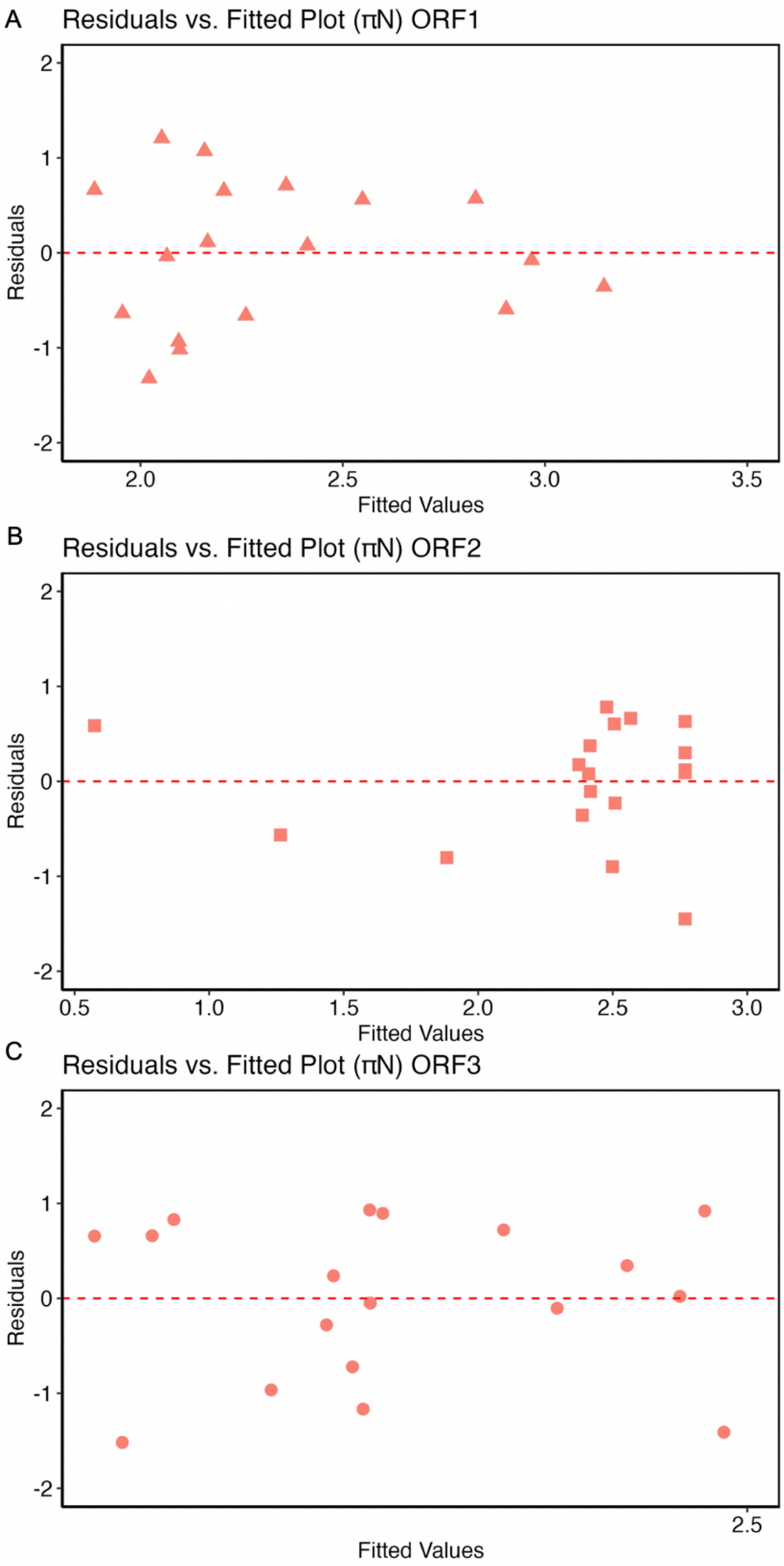

Supplementary Figure 3. The residuals versus fits plot for the data sets of nonsynonymous nucleotide diversity (π_N) and Log_{10} Reduction Value (LRV) in ORF1 **(A)**, ORF2 **(B)**, and ORF3 **(C)**

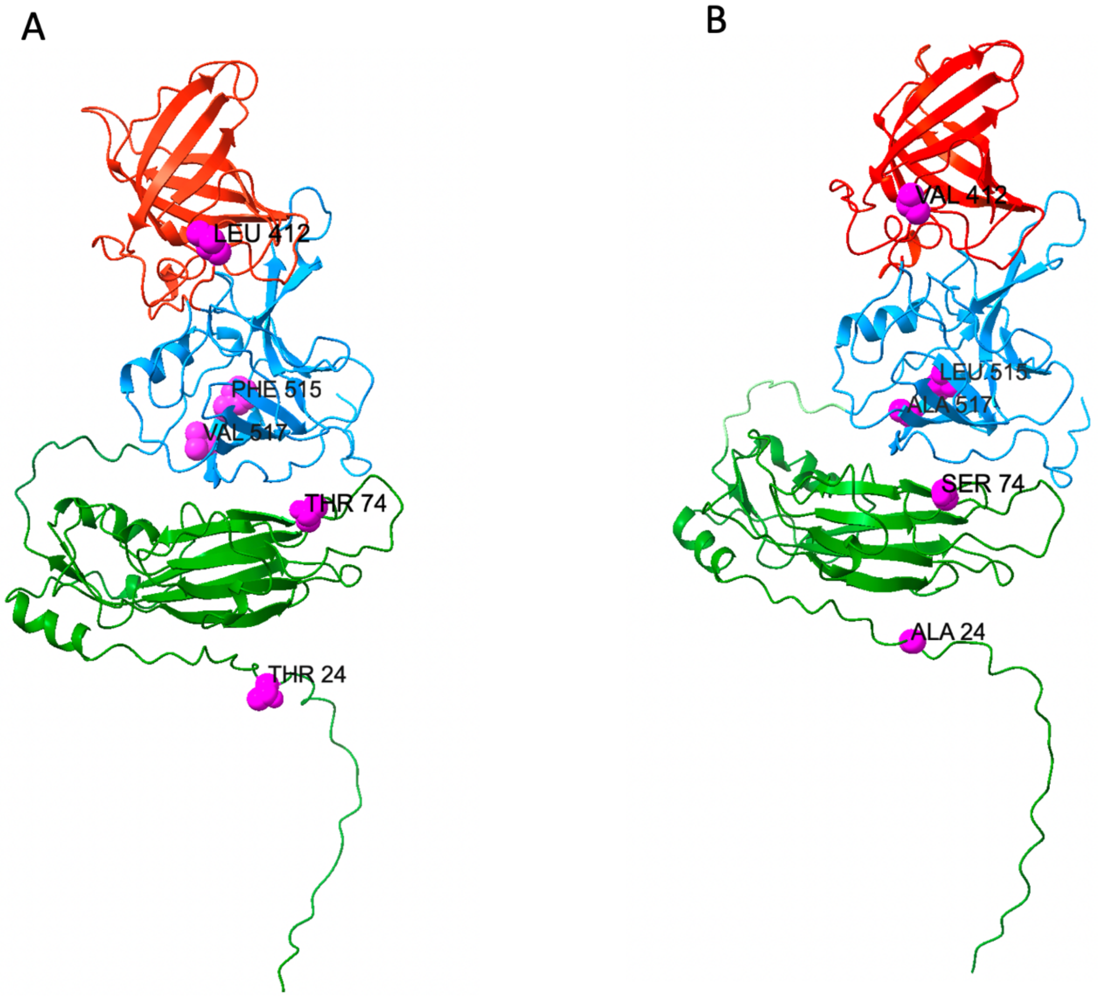

Supplementary Figure 4. The mapping of nonsynonymous mutations on VP1 in susceptible (A) and less susceptible (B) MNV populations. S is shaded green, P1 is shaded blue, P2 is shaded orange, and amino acid substitutions in VP1 are highlighted by purple balls which cause amino acid alteration from threonine (THR) to alanine (ALA), threonine (THR) to serine (SER), leucine (LEU) to valine (VAL), phenylalanine (PHE) to leucine (LEU), and valine (VAL) to alanine (ALA) (T24A, T74S, L412V, F515L, and V517A)

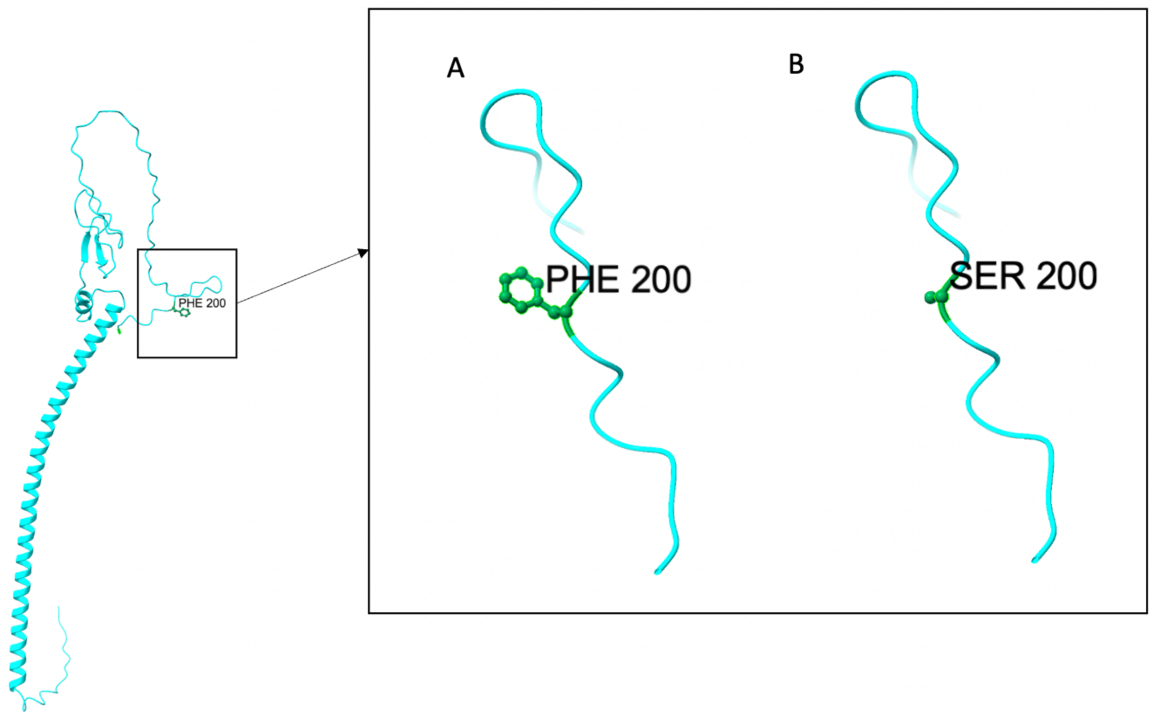

Supplementary Figure 5. The predicted secondary structure of the VP2 (cyan) with mapping nonsynonymous mutation (green) (phenylalanine (PHE) to serine (SER), of MNV populations before (A) and after (B) chlorine exposures

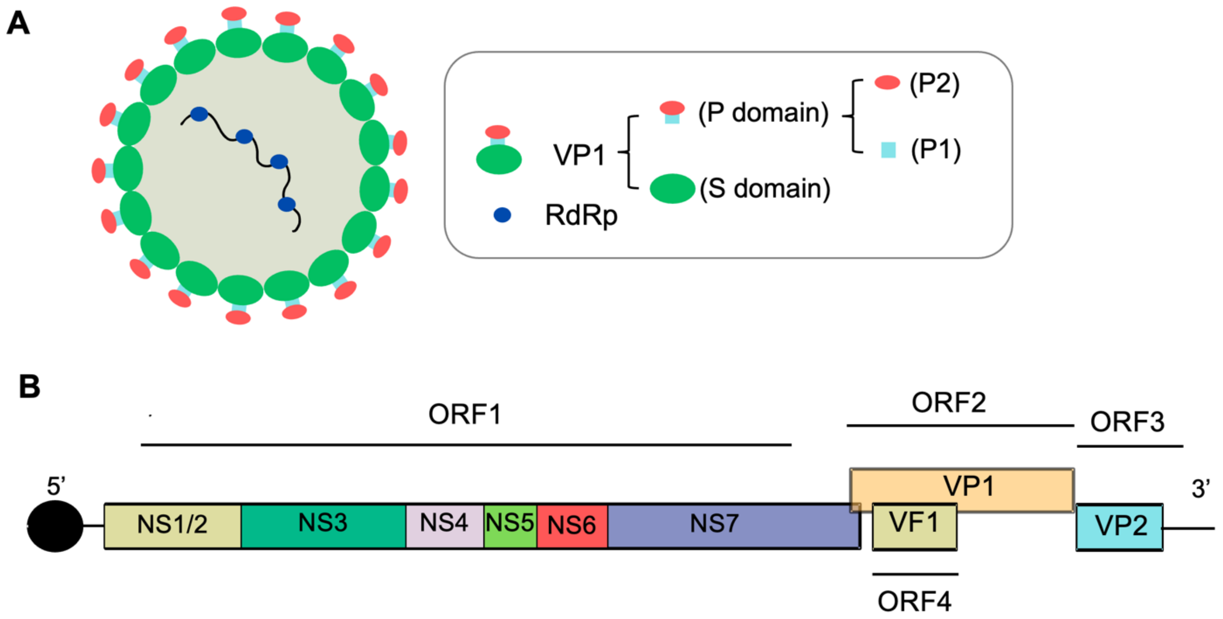

Supplementary Figure 6. Schematic diagram showing MNV genome organization (Zhang et al., 2023). The viral capsid consists of VP1, where the shell (S) domain surrounds the viral RNA, and the protruding (P) domain which divided into P1 and P2 subdomains. The RNA-dependent RNA polymerase (RdRp) is a key enzyme responsible for norovirus RNA replication (Deval et al., 2017) **(A)**. MNV has four ORFs, which encode six nonstructural proteins, major capsid VP1, minor capsid VP2, and virulence factor 1 (VF1), respectively **(B)** (Sosnovtsev et al., 2006; Barron et al., 2011; Zhang et al., 2023)

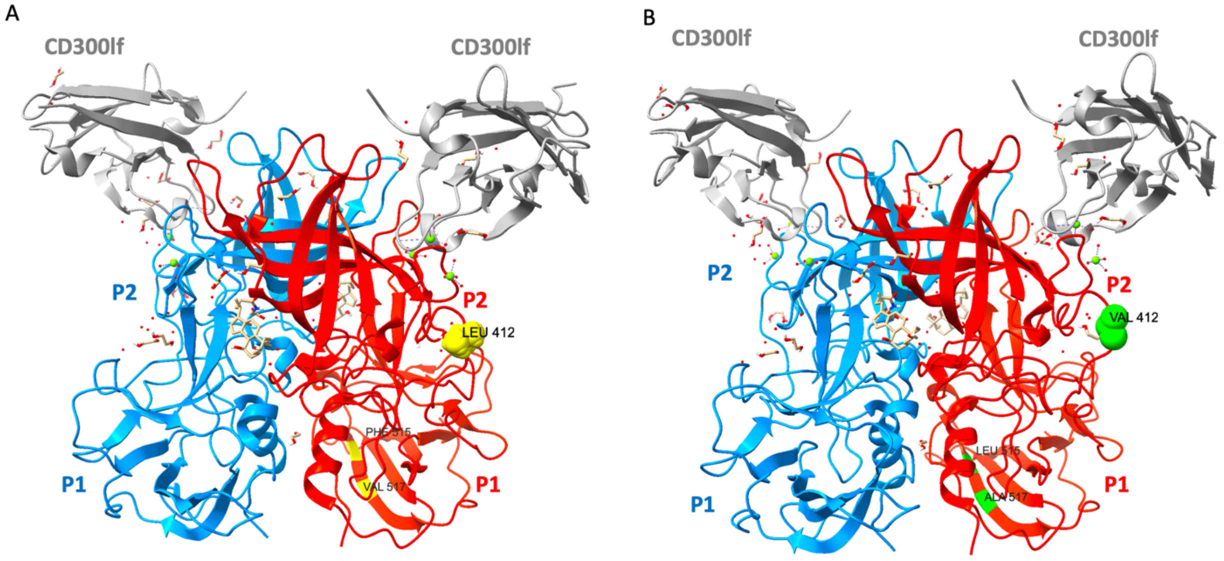

Supplementary Figure 7. Ribbon model of the CD300lf-P domain complex structure (Nelson et al., 2018). The two chains of the P domain dimer are blue and red, with the receptor protein CD300lf shown in silver. The amino acid substitutions in the P domain, which were L412V, F515L, and V517A before substitution, were represented as yellow spheres (left) and after substitutions, represented as green spheres (right) on the ribbon model.

Supplementary Table 1. Nucleotide and amino acid change in the MNV populations in first (A) and second rounds (B)

A

Coding Region	Round 1		
	Nucleotide Position	Amino Acid Change	Amino Acid Position
ORF1	T78C	S → P	25
	T561C	P → S	186
	A733G	Y → C	243
	C829T	T → M	275
	T2523C	S → P	840
ORF2	A5125G	T → A	24
	C5370T	No change	
	C5463T	No change	
	C6048T	No change	
	A6092G	K → R	346
	C6191T	T → M	379
	G6247A	V → I	398
ORF3	C6703T	A→ V	8
	C6748T	T → I	23
	G7088A	No change	
	T7215C	W → R	179
	T7279C	F → S	200
	C7358T	No change	

Note: A, alanine; R, arginine; C, cysteine; I, isoleucine; K, lysine; M, methionine; F, phenylalanine; P, proline; S, serine; T, threonine; W, tryptophan; Y, tyrosine; V, valine.

B

Coding Region	Round 2		
	Nucleotide Position	Amino Acid Change	Amino Acid Position
ORF1	T78C	S → P	25
	G138A	A → T	45
	T561C	P → S	186
	A711G	I → V	236
	A733G	Y → C	243
	C829T	T → M	275
ORF2	C4178T	No change	
	T4691A	No change	
	A5125G	T → A	24

	C5276G	T → S	74
	C5370T	No change	
	C6191T	T → M	379
	G6289C	L → V	412
	T6598C	F → L	515
	C6605T	V → A	517
ORF3	T7215C	W → R	179
	T7279C	F → S	200

Note: A, alanine; R, arginine; C, cysteine; I, isoleucine; L, leucine; K, lysine; M, methionine; F, phenylalanine; P, proline; S, serine; T, threonine; W, tryptophan; Y, tyrosine; V, valine.

References

Barron, E. L., Sosnovtsev, S. V., Bok, K., Prikhodko, V., Sandoval-Jaime, C., Rhodes, C. R., et al. (2011). Diversity of Murine Norovirus Strains Isolated from Asymptomatic Mice of Different Genetic Backgrounds within A Single U.S. Research Institute. *PLoS One* 6. doi: 10.1371/journal.pone.0021435

Deval, J., Jin, Z., Chuang, Y. C., and Kao, C. C. (2017). Structure(s), Function(s), and Inhibition of the RNA-dependent RNA Polymerase of Noroviruses. *Virus Res* 234, 21–33. doi: 10.1016/j.virusres.2016.12.018

Nelson, C. A., Wilen, C. B., Dai, Y. N., Orchard, R. C., Kim, A. S., Stegeman, R. A., et al. (2018). Structural Basis for Murine Norovirus Engagement of Bile Acids and the CD300lf Receptor. *Proc Natl Acad Sci U S A* 115, E9201–E9210. doi: 10.1073/pnas.1805797115

Sosnovtsev, S. V., Belliot, G., Chang, K.-O., Prikhodko, V. G., Thackray, L. B., Wobus, C. E., et al. (2006). Cleavage Map and Proteolytic Processing of the Murine Norovirus Nonstructural Polyprotein in Infected Cells. *J Virol* 80, 7816–7831. doi: 10.1128/jvi.00532-06

Zhang, Q., Zhu, S., Zhang, X., Su, L., Ni, J., Zhang, Y., et al. (2023). Recent Insights into Reverse Genetics of Norovirus. *Virus Res* 325. doi: 10.1016/j.virusres.2023.199046
